# Substantial rearrangements, single nucleotide frameshift deletion and low diversity in mitogenome of *Wolbachia*-infected strepsipteran endoparasitoid in comparison to its tephritid hosts

**DOI:** 10.1038/s41598-021-04398-y

**Published:** 2022-01-10

**Authors:** Sharon Towett-Kirui, Jennifer L. Morrow, Markus Riegler

**Affiliations:** Hawkesbury Institute for the Environment, Western Sydney University, Locked Bag 1797, Penrith, NSW 2751 Australia

**Keywords:** Entomology, Coevolution, Evolutionary genetics, Molecular evolution, Genomics

## Abstract

Insect mitogenome organisation is highly conserved, yet, some insects, especially with parasitic life cycles, have rearranged mitogenomes. Furthermore, intraspecific mitochondrial diversity can be reduced by fitness-affecting bacterial endosymbionts like *Wolbachia* due to their maternal coinheritance with mitochondria. We have sequenced mitogenomes of the *Wolbachia*-infected endoparasitoid *Dipterophagus daci* (Strepsiptera: Halictophagidae) and four of its 22 known tephritid fruit fly host species using total genomic extracts of parasitised flies collected across > 700 km in Australia. This halictophagid mitogenome revealed extensive rearrangements relative to the four fly mitogenomes which exhibited the ancestral insect mitogenome pattern. Compared to the only four available other strepsipteran mitogenomes, the *D. daci* mitogenome had additional transpositions of one rRNA and two tRNA genes, and a single nucleotide frameshift deletion in *nad5* requiring translational frameshifting or, alternatively, resulting in a large protein truncation. *Dipterophagus daci* displays an almost completely endoparasitic life cycle when compared to Strepsiptera that have maintained the ancestral state of free-living adults. Our results support the hypothesis that the transition to extreme endoparasitism evolved together with increased levels of mitogenome changes. Furthermore, intraspecific mitogenome diversity was substantially smaller in *D. daci* than the parasitised flies suggesting *Wolbachia* reduced mitochondrial diversity because of a role in *D. daci* fitness.

## Introduction

Animal mitochondrial genomes (mitogenomes) are double-stranded DNA molecules with a length of 15–18 kb. They are generally circular chromosomes consisting of 37 genes including 13 protein-coding genes (PCGs), 22 transfer RNA (tRNA) genes and two ribosomal RNA (rRNA) genes, and one AT-rich region, also known as the control region^1,2^. Mitochondrial genes and mitogenomes have widely been used for DNA barcoding, and in phylogenetic and phylogeographic analyses across many animal taxa because of their conserved function yet relatively high substitution rates, maternal inheritance and very low levels of recombination^3–5^. Mitogenome studies focussing on individual species have revealed that some species have very low mitogenome diversity, and this has generally been attributed to bottleneck effects, also known as founder effects^6,7^. In insects, reduced mitogenome diversity can also be caused by maternally inherited bacterial endosymbionts such as *Wolbachia* that can invade host populations by either manipulating host reproduction or increasing host fitness in other ways (for instance, increased fecundity or resistance against pathogens)^8,9^, resulting in selective sweeps and the hitchhiking of coinherited mitogenome variants^10–12^. Conversely, a recent modelling study also suggested that selection on mitochondrial genomes can lead to reduced symbiont variation across host populations^13^.

Comparative mitogenome analyses across multiple phylogenetically diverse insect taxa have revealed in some insect lineages unusual genome characteristics such as gene duplications, changes of gene order, indels and differences in codon usage, nucleotide content and secondary structures of tRNA genes^14–17^. The rearrangement of gene order can include transposition, inversion and inverse transposition of mitochondrial genes, and can be used to infer phylogenetic relationships across different taxonomic levels^18–20^. It has been hypothesised that mitogenome rearrangements may occur because of recombination, but recombination in animal mitogenomes is generally rare^21^. The more likely process may be tandem duplication of a set of genes followed by the random loss of a part of the duplication, also known as tandem duplication random loss (TDRL) events^22–24^. Mitochondrial gene duplications have been observed in the scorpion fly, ﻿*Microchorista philpotti*^25^ and in other invertebrates, such as *Leptotrombidium* chigger mites^26^ and the parasitic nematode, ﻿*Camallanus cotti*^27^. However, such duplications may not persist for long before they result in pseudogenisation and loss of duplicated genes, and are not frequently seen in lineages with rearranged mitogenomes^22–24^. Mitogenome fragmentation has also been observed in several arthropod taxa and other organisms, and can lead to mitogenomes consisting of several small circular chromosomes^28–30^.

Mitogenome rearrangements have occurred in several insect and other arthropod lineages with parasitic life cycles, for example, some hymenopteran endoparasitoid taxa^31^. Mitogenome rearrangements have also been found in ectoparasites, such as the wallaby louse, *Heterodoxus macropus,* and the small pigeon louse, *Campanulotes bidentatus compar*^20,32^. Similarly, mitogenome fragmentation has been found in parasitic human lice, *Pediculus humanus, Pediculus capitis* and *Pthirus pubis*^30^, the macaque louse, *Pedicinus obtusus* and the colobus louse, *Pedicinus badii*^29^.

Strepsiptera is a small insect order, with approximately 630 described species^33^. They are thought to have small genomes; using flow cytometry, the genome sizes of *Caenocholax fenyesi* and *Xenos vesparum* were estimated at 108 Mb and 130 Mb^34^. Their small genome size may be attributed to their endoparasitic life cycle, unusual morphological characteristics and unique features^18,35,36^. Strepsiptera display extreme sexual dimorphism. Adult females of most strepsipteran species are neotenic, with fused head and thorax, lacking typical characteristics of adult insects like wings, antennae, mouth and legs, and are permanently endoparasitic, except for the free-living adult females of the Mengenillidae^37,38^. In contrast, adult strepsipteran males undergo complete metamorphosis, and are free-living and winged^37^. Strepsiptera comprises two suborders, the Mengenillidia, with one family (Mengenillidae) and the Stylopidia with eight families, including the Xenidae and the Halictophagidae^33,39^. Strepsiptera are endoparasitoids of a wide range of hosts across seven insect orders: Blattodea, Hemiptera, Hymenoptera, Diptera, Mantodea, Orthoptera and Zygentoma^36,37^. Host attack occurs by the free-living first instar larvae (planidia). After three more larval instars within their hosts, in Stylopidia the neotenic females and male pupae extrude through the host’s cuticle; adult males then emerge from the pupae in the extrusions while the neotenic females remain fully endoparasitic. In contrast, Mengenillidae have maintained the ancestral state of free-living adults, and both females and males undergo pupation outside the host^36^.

Until recently, the phylogenetic placement of Strepsiptera and its species has proved to be a challenge due to their morphological peculiarities and the scarcity of molecular data^39–41^. The sequencing of the mitogenomes of two species each of *Mengenilla* (Mengenillidae) and *Xenos* (Xenidae) has provided substantial progress^18,42–44^. Mitogenome comparisons revealed more changes in *Xenos vesparum* than *Mengenilla australiensis* when compared to the inferred ancestral holometabolan mitogenome arrangement^18^. These additional changes arose with the transition from Mengenillidae which still leave the host for pupation and have free-living adult females and males, to Stylopidia with endoparasitic neotenic females and free-living adult males and, therefore, a more extreme endoparasitic strepsipteran life cycle^18^. Nevertheless, molecular data of the largest strepsipteran family, the Halictophagidae, are crucial for a more comprehensive understanding of strepsipteran evolution, and in particular, their interactions with hosts and the transition to the more extreme endoparasitic life cycle of Stylopidia, with males that pupate inside the host and neotenic females that are fully endoparasitic.

*Dipterophagus daci* is the only described strepsipteran parasitising Diptera (except for undescribed strepsipteran species from Papua New Guinean platystomatid flies) and has been recorded in 22 dacine fruit fly species (Tephritidae: Dacini) in Australia and the Solomon Islands^45–47^. Recent molecular analyses of whole genome sequencing (WGS) libraries of field-collected adult tephritid fruit flies from Australia detected genomic sequences of *D. daci*, including its entire mitogenome, indicative of concealed parasitisation of the sequenced flies^47^. Phylogenetic analyses of the *D. daci* mitochondrial *cox1*, *nad1*, *16S rRNA* and nuclear *18S rRNA* genes revealed that it belongs to the family Halictophagidae^47^, confirming earlier morphological analyses which placed it into the halictophagid subfamily Dipterophaginae^33,37^. The WGS analyses and further diagnostic testing of both parasitised and unparasitised tephritid fruit fly individuals revealed a clear link between *D. daci* and two *Wolbachia* strain sequence types, ST-285 and ST-289, previously detected in these tephritid fruit fly samples at low prevalence^48,49^; this demonstrated that *D. daci* is the true host of these two strains, *w*Ddac1 and *w*Ddac2, which occur at a high prevalence in *D. daci*^47^. Furthermore, no *Wolbachia* genes known to cause host reproductive manipulations were found, and there was a low diversity in the mitochondrial PCGs of *D. daci* when compared with its nuclear 18S rRNA gene sequences^47^. This suggests that due to its maternal coinheritance with mitochondria, *Wolbachia* may have reduced mitochondrial diversity as a consequence of a positive fitness effect on *D. daci*. However, it has not been analysed whether the extent of intraspecific mitogenome diversity differs between *D. daci* and its fruit fly host species, yet this may provide further evidence that *D. daci* is the actual host of *Wolbachia* rather than the fruit flies.

The hosts of *D. daci* include several dacine fruit fly species that are destructive pests of fruits and vegetables, for example *Bactrocera tryoni* (Queensland fruit fly, Australia’s most significant horticultural pest), its sibling species *Bactrocera neohumeralis*, *Bactrocera frauenfeldi*^50^ and many other species that are not major pests such as *Zeugodacus strigifinis* which develops in flowers of Cucurbitaceae^51,52^. Several Dacini mitogenomes have previously been sequenced, including of *B. tryoni*^53^, however, the mitogenomes of *B. frauenfeldi*, *B. neohumeralis* and *Z. strigifinis* have not yet been sequenced and characterised.

In this study, we obtained six mitogenome variants of *D. daci* and nine mitogenome variants of four of its 22 tephritid host species, *B. frauenfeldi*, *B. neohumeralis*, *B. tryoni* and *Z. strigifinis* by WGS of DNA libraries obtained from parasitised individual hosts. We then compared the arrangement, nucleotide composition and codon usage of these mitogenomes together with the previously sequenced mitogenomes of four other strepsipterans, four species of closely related insect orders and the host fruit flies. As mitogenome rearrangements have previously been detected in other strepsipterans^18,42–44^, we expected that the *D. daci* mitogenome would also differ from the ancestral mitogenome arrangement of insects and the fruit fly mitogenomes. Furthermore, we anticipated that *D. daci* mitogenomes contain more rearrangements compared to the mitogenomes of *Mengenilla* (with free-living adults) but share some of these differences with the mitogenomes of *Xenos* (with more extreme endoparasitic life cycles)*.* Furthermore, we compared the intraspecific mitogenome diversity between *D. daci* and the fruit flies from which the *D. daci* mitogenomes were obtained. Due to the previously described association of *D. daci* with *Wolbachia*^47^, we expected that intraspecific mitogenome diversity would be less in *D. daci* than the fruit fly species.

## Results

### Genome sequencing and assembly

**Figure 1:**
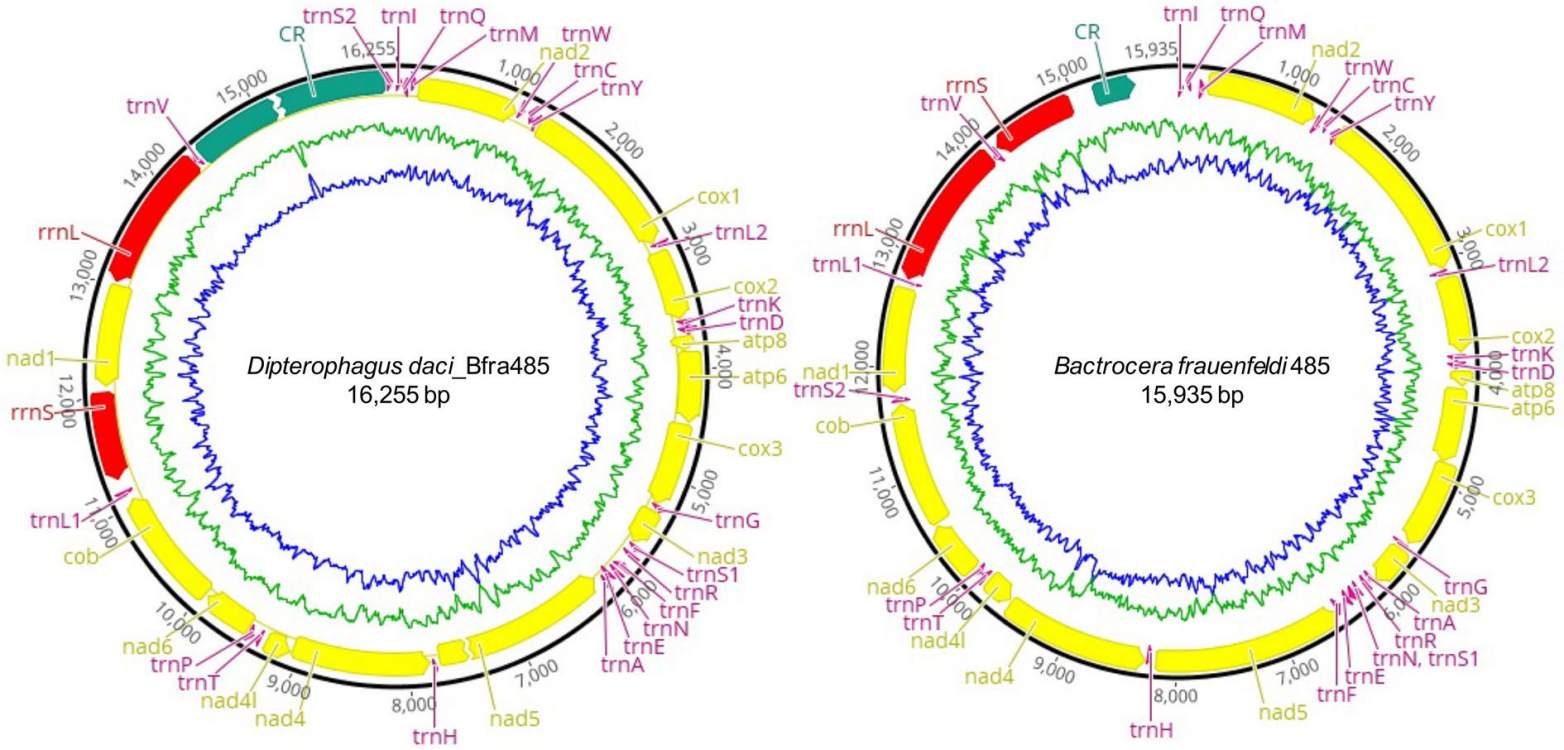
Structure of the mitogenomes of *Dipterophagus daci* and *Bactrocera frauenfeldi* obtained from a whole genome sequencing library of the genomic extract of the parasitised specimen *B. frauenfeldi* Bfra485. PCGs are denoted in yellow, rRNA genes in red, tRNA genes in purple and control region in green. The AT content (blue) and GC content (green) were plotted as the deviation from the average AT and GC content of the overall sequence using sliding window analysis. The mitogenome of *D. daci* has not been closed and contains one unusual single nucleotide frameshift deletion in the *nad5* gene.

Whole genome sequencing was performed on genomic extracts of nine individuals of four tephritid fruit fly species that were parasitised with *Wolbachia*-infected *D. daci* and were collected across a range of > 700 km in Australia (Table 1). Of these, six sequence libraries produced a good coverage (≥ 26.7-fold) of *D. daci* mitogenomes; three other sequence libraries contained *D. daci* mitogenomic sequences but not of sufficient coverage to assemble mitogenomes (Table 1). However, all nine sequence libraries included mitogenomes of the four fruit fly species. The *D. daci* and fruit fly mitogenomes were first filtered from the contig list of Bfra485 which had the highest read number. Its *D. daci* mitogenome comprised two contigs of approximately 12 kb and 3.2 kb while the fly mitogenome comprised one contig of approximately 15.9 kb. Then, iterative mapping using Bfra485 reads resulted in an almost complete *D. daci* mitogenome with a minimum estimated length of 16,255 bp and a complete circular *B. frauenfeldi* mitogenome of 15,935 bp (Fig. 1, Table S1). These two mitogenomes were used to filter the *D. daci* and fruit fly mitogenomes from the other sequence libraries. *Dipterophagus daci* mitogenomes were successfully assembled from six libraries (Bfra485, Bn171, Bn342, Bt194, Bt210 and Zst503). Albeit detectable, *D. daci* coverage in the three remaining libraries (Bn135, Bn240 and Bn244) was too low (< fivefold) for mitogenome assembly but was sufficient in Bn240 for the calling of single nucleotide polymorphisms (SNPs) at informative sites. The fruit fly mitogenomes were successfully assembled from all nine libraries (Bfra485, Bn135, Bn171, Bn240, Bn244, Bn342, Bt194, Bt210 and Zst503) (Table 1). The size of the mitogenomes ranged from 16,243 to 16,255 bp for *D. daci*, and from 15,858 to 15,935 bp for the fruit flies (Table S1).

**Table 1:** Summary of nine fruit fly WGS libraries obtained from individuals of four tephritid fruit fly species parasitised by *Dipterophagus daci*, collection localities, *Wolbachia* infection status (+ or −) with *w*Ddac1 (ST-285) and *w*Ddac2 (ST-289), number of reads after QC and coverage for the *D. daci* and fruit flies mitogenomes. Mitogenomes with high coverage are presented in bold, with coverage number in parentheses. All nine fruit fly samples contained *D. daci* as detected by sequence reads and PCR^47^.

Tephritid species	Sample ID	Collection locality	*w*Ddac1 (ST-285)	*w*Ddac2 (ST-289)	Number of reads after QC	*D. daci* mitogenome mapped reads (coverage)	Fly mitogenome mapped reads (coverage)
*Bactrocera frauenfeldi*	485	Cairns	y	y	109,057,960	**15,938 (104.5)**	**11,544 (72)**
*Bactrocera neohumeralis*	135	Mourilyan Harbour	y	y	68,308,764	6 (0.04)	**86,7084 (5,427)**
*Bactrocera neohumeralis*	171	Townsville	y	y	77,482,368	**4094 (26.8)**	**62,060 (387)**
*Bactrocera neohumeralis*	240	Mourilyan Harbour	y	n	72,186,748	320 (2.1)	**36,332 (228)**
*Bactrocera neohumeralis*	244	Cairns	n	y	60,128,324	52 (0.3)	**496,912 (3101)**
*Bactrocera neohumeralis*	342	Mackay	y	y	67,282,474	**4086 (26.7)**	**47,976 (300)**
*Bactrocera tryoni*	194	Cairns	y	y	79,574,356	**8104 (53.2)**	**530,836 (3312)**
*Bactrocera tryoni*	210	Mackay	y	y	63,859,882	**6892 (45.2)**	**18,486 (115.4)**
*Zeugodacus strigifinis*	503	Cairns	y	y	65,468,734	**11,210 (73.5)**	**579,200 (3646)**

### Mitogenome structure

The six *D. daci* and nine fruit fly mitogenomes each contained 13 PCGs, two rRNA genes and 22 tRNA genes (Table S2). In the *D. daci* and fruit fly mitogenomes, nine PCGs (*nad2, cox1, cox2, atp8, atp6, cox3, nad3, nad6* and *cob*) and 14 tRNA genes (*trnI, trnM, trnW, trnL*_*2*_*, trnK, trnD, trnG, trnS*_*1*_*, trnR, trnN, trnE, trnA, trnT* and *trnS*_*2*_) were located on the major strand (leading strand) while four PCGs (*nad5, nad4, nad4L* and *nad1*), eight tRNA genes (*trnQ, trnC, trnY, trnF, trnH, trnP, trnL*_*1*_ and *trnV*) and both rRNA genes (*rrnL* and *rrnS*) were located on the minor strand (lagging strand) (Fig. 1, Table S2). The AT-rich region of the fruit fly mitogenomes was located between *rrnS* and *trnI* and had an average length of 594 bp, while in *D. daci* the AT-rich region was located between *trnV* and *trnS*_*2*_. Furthermore, the *D. daci* mitogenomes contained an unresolved sequence assembly gap between *trnV* and *trnS*_*2*_ resulting in variable lengths (Table S2)*.*

### Mitogenome base composition

**Figure 2:**
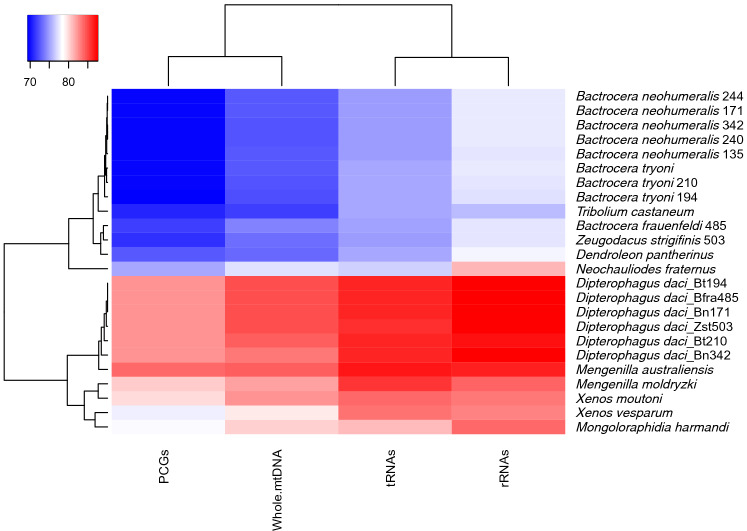
Comparative analysis of AT content of mitogenomes of *Dipterophagus daci*, its host fruit fly species and other reference species.

The nucleotide composition of *D. daci* mitogenomes was AT-biased (approximately 84%) and this was similar to the mitogenomes of the other strepsipterans. The fruit fly mitogenomes were less AT-biased (approximately 72%) (Fig. 2, Table S1) and their AT contents were similar except for *B. frauenfeldi* 485 and *Z. strigifinis* 503, which had AT contents of 74.1% and 73.4% respectively (Fig. 2, Table S1). Comparative mitogenome analyses of *D. daci* and their fruit fly hosts revealed a clear bias in nucleotide composition with positive AT-skews and negative GC-skews (Table S1). This was also noted for *X. vesparum* while *M. australiensis* and *Mengenilla moldryzki* had a negative AT skew (Table S1). All the insect taxa had a negative GC skew (Table S1).

### Mitochondrial protein coding genes

The total length of the 13 PCGs of the *D. daci* mitogenomes was on average 10,696 bp and was relatively shorter than the total length of the PCGs of the fruit fly mitogenomes with an average length of 11,187 bp (Table S1). The start codons ATT, ATA and ATG were used in both *D. daci* and fruit fly PCGs, except the fruit fly *atp8* gene which started with GTG (Table S2). In *D. daci* PCGs *nad1*, *nad2, nad3* and *nad4L* started with ATA, *cox2, atp8*, *nad5* and *nad6* with ATT, *atp6, cox3, nad4* and *cob* with ATG, and *cox1* with CAA (Table S2). Furthermore, the *D. daci* PCGs *nad2, atp8, nad6, cox3, nad4L* and *nad1* ended with TAA, while it is assumed that the remaining PCGs that ended with T are completed by adding 3’ A nucleotides to the mRNA (Table S2)*.*

The fruit fly PCGs *nad2, nad3, nad5* and *nad6* started with ATT, *cox2, atp6, cox3, nad4, nad4L* and *cob* with ATG, *atp8* with GTG, *nad1* with ATA, and *cox1* with TCG (Table S2). Seven fruit fly PCGs stopped with TAA, while *nad3* and *nad4* stopped with TAG; *nad5*, *cob* and *nad1* that ended with T, (and *cox1* ending with TA) are presumably completed by adding 3’ A nucleotides to the mRNA (Table S2). Comparative analyses of the relative synonymous codon usage (RSCU) revealed that across *D. daci*, the fruit fly and the other insect species, codons ending with A or T prevailed. Amino acids Ala, Gly, Leu, Pro, Arg, Ser, Thr and Val were commonly used, and Leu had the highest RSCU in all insect species (Table S3).

**Figure 3:**
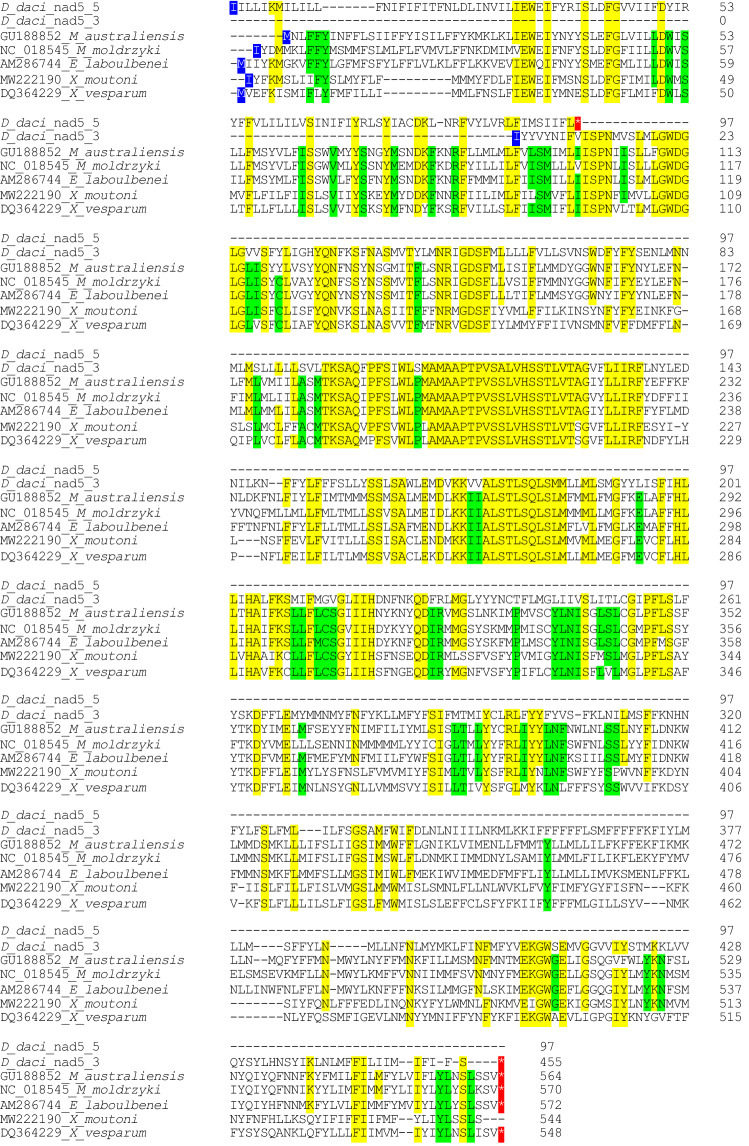
Amino acid (aa) alignment of the *nad5* gene of *Dipterophagus daci* (Bfra485) and five strepsipteran species, *Mengenilla australiensis*, *Mengenilla moldryzki*, *Eoxenos laboulbenei* (Mengenillidae), *Xenos vesparum* and *Xenos cf. moutoni* (Xenidae), listed with their GenBank accession numbers*.* The red-highlighted star indicates stop codons, including a stop codon at position 98 in *D. daci*, with a new start codon (highlighted in blue) upstream of the mutation. Positions with > 0.5 conserved aa across sequences are highlighted in yellow when *D. daci* displays the conserved aa, or green when *D. daci* is different; the 5’ sequence of *D. daci* reads from an alternative open reading frame starting position than the 3’ sequence due to the deletion that inserts a stop codon.

Surprisingly, the *nad5* gene contained an unusual deletion of one nucleotide (nucleotide position 291) in all six *D. daci* mitogenomes which introduced an in-frame stop codon (TAA) at amino acid position 98 (Fig. 3); the remainder of *nad5* further downstream, however, still constituted an open reading frame but started from a different position. The unexpected finding of a single nucleotide -1 frameshift deletion was further verified by Sanger sequencing of the *nad5* region of *D. daci* from five samples in addition to those used for WGS; these samples did not undergo REPLI-g amplification which was used for the WGS samples prior to library preparation (Table S4). All *nad5* gene Sanger sequences were identical to the assembled mitogenomes and confirmed this nucleotide deletion. Subsequently, the domain architecture of the *nad5* gene was checked using CDART (NCBI)^54^. This revealed that, similar to other *nad5* genes, the second part of the *D. daci nad5* gene downstream of the deletion contained the proton-conducting transporter domain starting at amino acid position ~ 100 in most full-length strepsipteran *nad5* genes (Fig. 3), suggesting that this larger fragment of *nad5* of *D. daci* could still encode for a functional yet truncated protein.

### Mitochondrial tRNA and rRNA genes

The *D. daci* and fruit fly mitogenomes contained 22 tRNA genes (Fig. 1, Table S2). Their average total length was 1424 bp in *D. daci* and 1468 bp in fruit fly mitogenomes (Table S1). Both 16S rRNA and 12S rRNA genes (*rrnL* and *rrnS* respectively), had a total length of 2074 bp in the *D. daci* mitogenomes, while both combined ranged from 2081 to 2110 bp in the fruit fly mitogenomes (Table S1). Across the six *D. daci* mitogenomes, MITOS2 could only identify one part (688 bp 3’ section adjacent to the *nad1* gene) of the 16S rRNA gene because the 5’ section flanked by *trnV* was highly diverged. However, the entire coding sequence was confirmed by sequence alignment with 16S rRNA genes of the reference strepsipteran mitogenomes obtained from GenBank and by BLASTn. In fruit fly mitogenomes the 16S rRNA gene was flanked by *trnL*_*1*_ and *trnV* and the 12S rRNA gene was flanked by *trnV* and the AT-rich region (Fig. 1, Table S2).

### Mitochondrial gene arrangement

**Figure 4:**
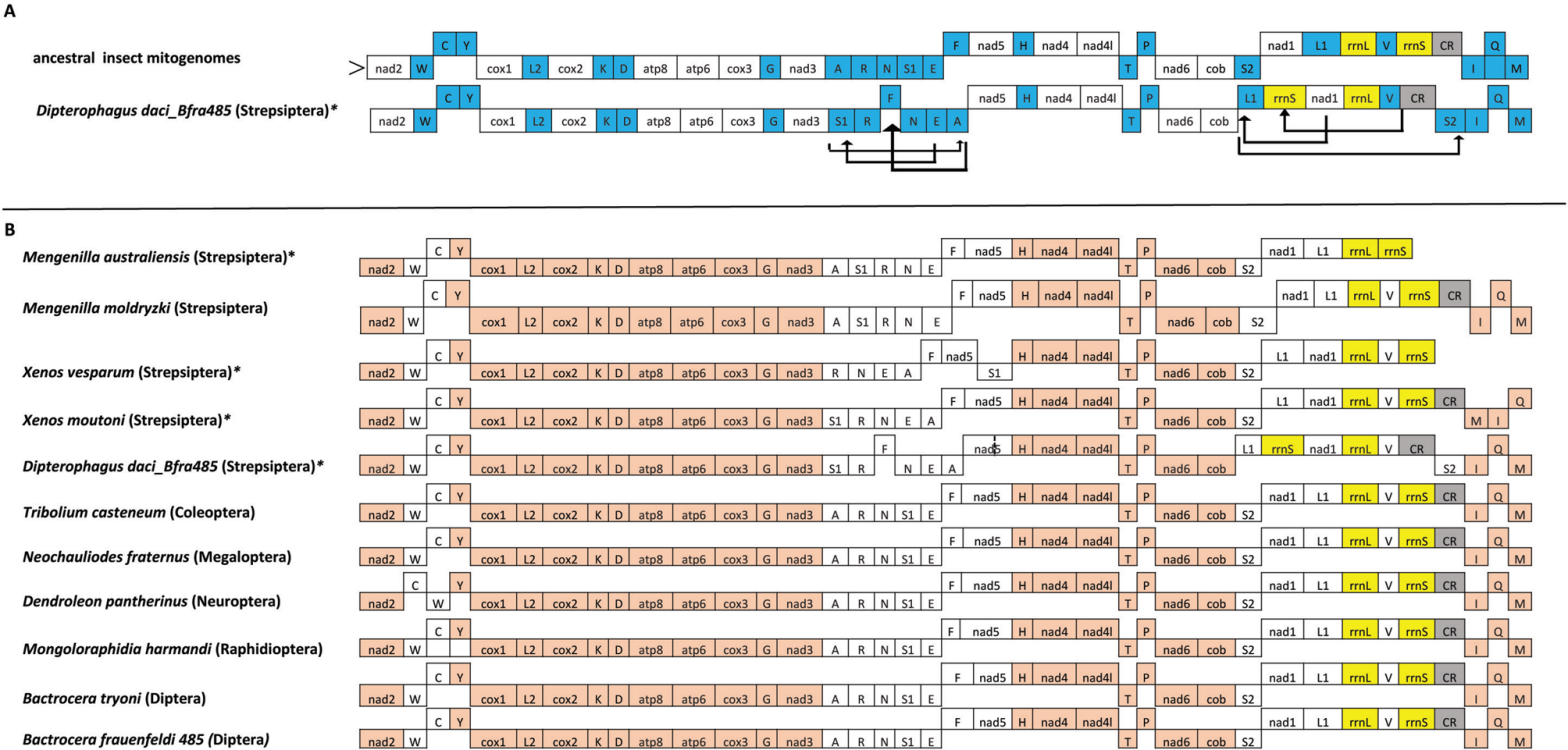
Organisation and rearrangement of the *Dipterophagus daci* mitogenome (**A**) compared to the ancestral holometabolan pattern; tRNA genes are blue, rRNA genes are yellow, protein coding genes are white and the control region is grey. The major (leading) strand is denoted by > and arrows denote gene translocations; (**B**) compared to ten other insect species (including four strepsipteran species and the host species *Bactrocera frauenfeldi* and *Bactrocera tryoni*); conserved gene arrangement (salmon colour) and different gene arrangements (white) in *D. daci* and the other species; the control region is grey*.* Mitogenome representation is not drawn to scale; * indicates species for which only incomplete mitogenomes are available.

Significant gene rearrangements were observed in the *D. daci* mitogenomes relative to the ancestral insect mitogenome, while the gene arrangement of the fruit fly mitogenomes were identical to the ancestral insect mitogenome pattern (Fig. 4A,B). Gene rearrangements in the *D. daci* mitogenomes were observed in two regions: the first region involved the transposition of *trnA*, *trnS*_*1*_ and *trnF*; and the second region involved the transposition of *trnS*_*2*_, *trnL*_*1*_ and *rrnS* (Fig. 4A), resulting in a different rRNA gene order when compared to all other mitogenomes.

**Figure 5:**
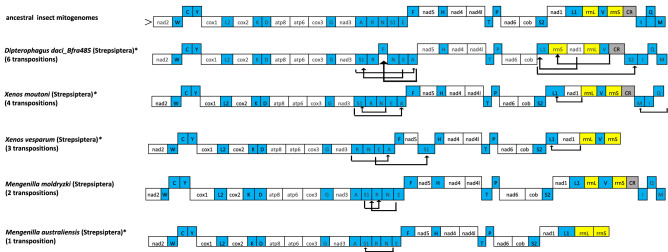
Mitogenome organisation and rearrangement illustrating gene translocations and number of transpositions in *Dipterophagus daci* and four strepsipteran species relative to the ancestral pattern in insect mitogenomes; tRNA genes are blue, rRNA genes are yellow, protein coding genes are white and the control region is grey. The major (leading) strand is denoted by > , arrows denote gene translocations and * indicate species for which only incomplete mitogenomes are available. Mitogenome representation is not drawn to scale.

The *D. daci* mitogenome arrangement was also compared with the mitogenomes of the four other strepsipteran species, one representative species each of four closely related insect orders (Coleoptera, Megaloptera, Neuroptera, Rhaphidioptera), *B. frauenfeldi* 485 and a reference *B. tryoni* (GenBank accession NC014611) (Fig. 4B). Generally, most genes in the *D. daci* mitogenome had a conserved gene arrangement position (Fig. 4B). However, comparisons revealed that *D. daci* contained more mitogenome rearrangements (6 transpositions) compared to *Xenos cf. moutoni, X. vesparum, M. moldryzki* and *M. australiensis* that contained 4, 3, 2 and 1 transpositions, respectively (Fig. 5). The transposition of *trnS*_*1*_ observed *in D. daci* was also observed in the four strepsipteran species, and the transposition of *trnA* and *trnL*_*1*_ was also found in *X. cf. moutoni* and *X. vesparum* (Fig. 5)*.* The transposition of *trnF*, *trnS*_*2*_ and *rrnS* were unique to *D. daci*, while the transposition of *trnM* (from I-Q-M in ancestral arrangement to M-I-Q) was unique to *X. cf. moutoni* and not seen in *D. daci* (Fig. 5). Mitogenomes of the fruit flies as well as the three representative species of Coleoptera, Megaloptera and Rhaphidioptera were arranged according to the ancestral insect mitogenome pattern while *Dendroleon pantherinus* (Neuroptera) exhibited a C-W-Y (W–C-Y in ancestral) gene arrangement (Fig. 4B).

### Intraspecific mitogenome variation

We performed multiple sequence alignments to investigate the intraspecific diversity across the six *D. daci* mitogenome variants from six sequence libraries, and also obtained informative SNP data from an additional library (Bn240) that had low *D. daci* mitogenome coverage but was sufficient for SNP calling of sites variable between the six mitogenomes. We identified a total of ten SNPs occurring in four mitochondrial PCGs, including *cox1, nad5, nad4* and *cob* (Table 2) and a total of 34 SNPs occurring in the *D. daci* mitogenome variants (Table S5). To contrast intraspecific mitogenome variation, we investigated the diversity of the 13 PCGs of the assembled six *D. daci*, five *B. neohumeralis* and two *B. tryoni* mitogenome variants obtained in this study and the reference *B. tryoni* mitogenome variant. Despite the relatively low mitogenome sample number, intraspecific nucleotide diversities were substantially lower in the PCGs of the *D. daci* mitogenome variants than in the PCGs of the fruit fly mitogenome variants (Table 3). In contrast to the ten SNPs in the mitochondrial PCGs of *D. daci*, the mitochondrial PCGs of *B. neohumeralis* and *B. tryoni* had 298 and 133 SNPs, respectively, showing that the PCGs of the *B. neohumeralis* and *B. tryoni* mitogenomes were 33.1 × and 14.7 × more diverse than the *D. daci* mitogenome (Table 3).

**Table 2:** Single nucleotide polymorphisms (SNPs) in protein coding genes of *Dipterophagus daci* mitogenomes, showing the collection locality, *Wolbachia infection* status (+ or −) with *w*Ddac1 (ST-285) and *w*Ddac2 (ST-289) and the SNP position in the mitogenome; * denotes the assembled reference mitogenome of *D. daci* from *Bactrocera frauenfeldi* Bfra485 (MW233588) and ^ denotes the library with low coverage that did not allow assembly of the mitogenome; empty cells indicate that the positions have the same nucleotide as the assembled reference genome.

Collection locality	*w*Ddac1	*w*Ddac2	Gene	*cox1*		*nad5*				*nad4*		*cob*	
			Nucleotide position in the mitogenome	1762	2546	6408	6607	6912	7306	7869	8640	10,276	11,033
**Cairns**	**y**	**y**	*Dipterophagus daci*_Bfra485*	**C**	**A**	**G**	**G**	**T**	**C**	**T**	**G**	**A**	**A**
Townsville	y	y	*Dipterophagus daci*_Bn171	T				C		C	A	C	G
Mourilyan Harbour	y	n	*Dipterophagus daci*_Bn240^						G	C	A	C	
Mackay	y	y	*Dipterophagus daci*_Bn342				A						
Cairns	y	y	*Dipterophagus daci*_Bt194			A							
Mackay	y	y	*Dipterophagus daci*_Bt210		G								
Cairns	y	y	*Dipterophagus daci_*Zst503			A

**Table 3:** Nucleotide diversity of the mitochondrial PCGs of *Dipterophagus daci* (n = 6), *Bactrocera neohumeralis* (n = 5) and *Bactrocera tryoni* (n = 3), showing the number of single nucleotide polymorphisms (SNPs). The 5’ part of the *D. daci nad5* gene with the stop codon is listed separately as *nad5_5’*.

Gene	*Dipterophagus daci* (n = 6) PCGs		*Bactrocera neohumeralis* (n = 5) PCGs		*Bactrocera tryoni* (n = 3) PCGs	
	Total number of nucleotides	SNPs	Total number of nucleotides	SNPs	Total number of nucleotides	SNPs
***atp6***	642	0	678	16	678	8
***atp8***	150	0	162	5	162	1
***cob***	1111	2	1135	23	1135	13
***cox1***	1507	2	1535	42	1535	12
***cox2***	652	0	690	18	690	8
***cox3***	768	0	789	17	789	9
***nad1***	942	0	940	24	940	9
***nad2***	927	0	1023	22	1023	10
***nad3***	343	0	354	10	354	5
***nad4***	1263	2	1341	45	1342	22
***nad4L***	264	0	291	7	297	1
***nad5***	1350	4	1720	55	1720	26
***nad5_5’***	291	0	na	na	na	na
***nad6***	486	0	525	14	525	9
**Total PCG**	10,696	10	11,183	298	11,190	133

## Discussion

We have analysed the mitogenome of *D. daci* as the first sequenced mitogenome of Halictophagidae, the largest strepsipteran family, together with the mitogenomes of four of its 22 tephritid fruit fly host species, *B. frauenfeldi*, *B. neohumeralis*, *B. tryoni* and *Z. strigifinis*. We obtained these sequences from fly individuals with concealed *D. daci* parasitisation. Mitogenome analyses revealed extensive mitogenome rearrangements in *D. daci* relative to the inferred ancestral holometaboloan mitogenome arrangement and the fruit fly mitogenomes. Furthermore, in comparison to the other strepsipteran mitogenomes, *D. daci* has, with six gene transpositions, the most re-arranged strepsipteran mitogenome characterised so far. While it shared some of the mitogenome rearrangements with other Strepsiptera, *D. daci* contained additional and unique mitogenome differences. These included a single nucleotide -1 frameshift deletion in the coding region of the *nad5* gene possibly requiring translational frameshifting^16,17^, other unknown compensation mechanisms, or, alternatively, leads to a significant truncation of the gene product. Another unusual feature was a different order of the rRNA genes because of the transposition of the *rrnS* gene. Our findings also revealed that *D. daci* mitogenomes have shorter PCGs than mitogenomes of other insects which is typical for strepsipterans^18,43^. Despite the low sample number but whole-mitogenomic representation and similar sampling effort for *D. daci* and fruit fly species across a geographic range of > 700 km, covering a large part of known *D. daci* distribution^45^, we observed substantially (15-33x) lower genetic diversity in the *D. daci* mitochondrial PCGs relative to their host fruit fly species, suggesting that *Wolbachia* may be the cause for the loss of mitogenome diversity in *D. daci*.

Insect mitogenomes have a fairly conserved gene order, however, gene rearrangements occur in several insect taxa^55^. In the current study, we found extensive gene rearrangements in *D. daci* mitogenomes relative to the ancestral holometabolan mitogenome pattern. Mitochondrial gene rearrangements are usually characterised by either transposition, inversion or inverse transposition^56^, and more frequently involve tRNA genes than PCGs and rRNA genes^57^. In *D. daci*, rearrangements involved six gene transpositions (five tRNA genes and one rRNA gene). These were more mitogenomic transpositions in *D. daci* than in any other strepsipterans further suggesting that the *D. daci* lineage has experienced accelerated structural mitogenome rearrangements. The transpositions of *trnF*, *trnS*_*2*_ and *rrnS* were unique to *D. daci,* however, the transpositions of *trnA* and *trnL*_*1*_ were also observed in *X. cf. moutoni* and *X. vesparum*, while the transposition of *trnS*_*1*_ was common to the five strepsipteran species.

We also found that *nad5* of *D. daci* had one single nucleotide -1 frameshift deletion that resulted in the introduction of a stop codon at amino acid position 98. However, the downstream part of the gene still had an open reading frame but starting with another nucleotide position. This could be indicative that *D. daci* experiences translational frameshifting, similar to the translational editing mechanism proposed to overcome the issues of single nucleotide insertion and deletions found in PCGs of some animal mitogenomes^58^. Previously, single nucleotide insertions have been observed in *cob* of ants^16^ and *nad3* of some bird and turtle species^17^. It is noteworthy that our finding is, to our knowledge, the first example of -1 frameshift deletion found in an invertebrate mitogenome. So far single nucleotide deletions in mitochondrial PCGs have only been found in a few turtle species^58^, and, overall, -1 frameshift deletions appear to be rarer than +1 frameshift insertions^59^. Alternatively, the single nucleotide deletion in *nad5* of *D. daci* could result in the expression of a truncated but still functional *nad5* gene product because it still contained the proton-conducting transporter domain similar to *nad5* genes in other species^60^, however, this scenario may be less likely because it would constitute a substantial truncation. Yet another scenario could be compensation of the frame shift mutation by an unknown mechanism other than translational frameshifting, via the *D. daci* nuclear genome, *Wolbachia* or the fruit fly mitochondrial or nuclear genomes. There are several examples of intracellular endosymbionts with degraded gene functions that are compensated by other endosymbionts^61^ or their hosts^62^.

It has previously been hypothesised that mitogenome rearrangements arose with the evolution of parasitic life cycles. This is because a transition to a parasitic life cycle in a lineage may come in hand with a relaxation of selective constraints acting on mitogenomes and their functions^40^. Based on our findings we can now add single nucleotide frameshift mutations that may also arise in lineages that have evolved parasitic life cycles. There is evidence for the association between mitogenome changes and evolution of parasitic life cycles, because mitogenomes of parasitic lineages of Hymenoptera are highly rearranged when compared to the conserved mitogenome arrangement patterns in the more basal lineages of Hymenoptera which are not parasitic^31^. Mitogenome rearrangements were also reported for the two egg parasitoids, *Trichogramma japonicum* and *Trichogramma ostriniae*^55^ as well as a parasitoid of *Drosophila* larvae, *Leptopilina boulardi*^63^*.* Similarly, rearrangements have been observed in three parasitoid wasp species of the genus *Psyttalia* which parasitise *Bactrocera oleae*^64^. Furthermore, the numbers of mitogenome rearrangements in Strepsiptera correlated with the transition from moderate to extreme levels of parasitism*.* More gene rearrangements were observed in the mitogenomes of the Stylopidia species *D. daci*, *X. cf. moutoni* and *X. vesparum* compared to the Mengenillidia species *M. australiensis* and *M. moldryzki*. The largest number of differences when compared to the ancestral insect mitogenome arrangement were observed in *D. daci*, and the single nucleotide frameshift deletion in *nad5* and the transposition of *rrnS* were unique, and possibly associated with the more extreme endoparasitism displayed by *D. daci* and its different host utilisation (i.e. Diptera). Rearrangements involving ribosomal RNA genes have been found in other insects, such as thrips^65^. It is unclear how the *nad5* frameshift deletion could have occurred, but its effect may not be as severe in an endoparasitic insect^36^. Flight muscles rely heavily on mitochondrial function^66,67^, and an insect with limited flight function may be able to cope with a less efficient mitochondrial function.

The overall length of the *D. daci* and fruit fly mitogenomes were within the expected length of 15–18 kb^3^. Both *D. daci* and the fruit fly mitogenomes contained the 37 genes and the AT-rich region usually found in animal mitogenomes^1,2^. The conserved location for AT-rich region is between *rrnS* and *trnI,* however in the *D. daci* mitogenome the AT-rich region is located between *trnV* and *trnS*_*2*_, which is similar to its position in a gnat bug, *Stenopirates* sp.^68^*,* while it is located in the conserved location in *M. moldrzyki*^42^ and *X. cf. moutoni*; however, incomplete information is available for *X. cf. moutoni*^44^*.* The *D. daci* mitogenome assembly contained a gap in this region and hence the full length of the AT-rich region could not be estimated. Attempts to close the mitogenome by iterative mapping with short reads proved impossible. This region could either be too long and repetitive to be closed with bioinformatics approaches, or have secondary folding structures resulting in sequencing difficulties, as also found for *M. australiensis*, *X. cf. moutoni* and *X. vesparum*^18,43,44^.

Our study revealed that the mitochondrial PCGs of *D. daci* are shorter relative to the PCGs of their host fruit flies, and this could be associated with the evolution of the strepsipteran life cycle, as also suggested for *M. australiensis*, *X. cf. moutoni* and *X. vesparum*^18,43,44^. Similar to other parasitic insects^3,55^, the nucleotide composition of the *D. daci* mitogenomes were more AT-biased compared to fruit fly mitogenomes. The high AT bias observed in *D. daci* was found to be similar to the other Strepsiptera^18,43,44^. Furthermore, the *D. daci* mitogenome had a positive AT skew and a negative GC skew indicating that its genes contain more A than T, and more C than G, as also reported for other insects^69^.

Low intraspecific mitogenome diversity is generally attributed to founder events^70,71^, or can be due to *Wolbachia* endosymbionts which manipulate host reproduction or provide a fitness benefit to hosts^9,12^. Maternal coinheritance of mitogenomes and *Wolbachia* may facilitate *Wolbachia*-driven selective sweeps of the infected mitochondrial haplotype resulting in low mitochondrial genetic diversity^10–12,72^. In comparison to *B. neohumeralis* and *B. tryoni*, *D. daci* mitogenomes had only ten SNPs in PCGs and were 15–33 × less diverse. Previously, it has been demonstrated that *D. daci* hosts two *Wolbachia* strains, *w*Ddac1 and *w*Ddac2; these two strains lack genes required for host reproductive manipulations, and therefore may have beneficial effects on host fitness^47^. Our extensive mitogenome analysis of *D. daci* together with the previous analysis of its nuclear 18S rRNA gene filtered from the WGS libraries provides strong evidence that the low diversity observed in the *D. daci* mitogenome could be due to a past *Wolbachia* invasion with hitchhiking mitogenome types. In addition, the detection of high prevalence of *Wolbachia* in *D. daci*^47^ also suggests that *Wolbachia* confers a fitness benefit to *D. daci*. It is unknown, however, whether both strains invaded this host at once, or in two separate waves. Further characterisation of *D. daci* genetic diversity and the *D. daci*-*Wolbachia* relationship across larger population samples will be required to further investigate *Wolbachia* effects on mitogenome diversity patterns and host fitness in this species.

## Methods

### Insect specimens and WGS

This study analysed WGS libraries of nine males of four tephritid fruit fly species (*B. frauenfeldi*, *B. neohumeralis*, *B. tryoni* and *Z. strigifinis*) representing field populations across a region from Mackay to Cairns (> 700 km distance) in Queensland, Australia (Table 1). These specimens formed part of a previous survey of *Wolbachia* in 24 Australian tephritid fruit fly species and were collected using traps with male attractants as previously described^48,49^. DNA was extracted from fly abdomens and tested for *Wolbachia* using *Wolbachia surface protein* (*wsp*) and 16S rRNA gene primers; furthermore, two strains of *Wolbachia*-positive flies were characterised using multi-locus sequence typing (MLST) as ST-285 and ST-289^48,49^ (Table 1), with later assignment of these strains to their actual host *D. daci* as *w*Ddac1 and *w*Ddac2^47^. DNA extracts of 14 *Wolbachia*-positive flies were selected and amplified by multiple displacement using the REPLI-g mini kit (Qiagen) previously used to amplify DNA of mitochondrial and bacterial chromosomes at higher coverage than eukaryotic chromosomes^11^ and submitted for library construction and WGS using the Illumina Hiseq2500 platform as previously described^47^. Nine of these 14 WGS libraries produced sufficient mitogenome coverage and were used for analyses in the current study (Table 1). The remaining five WGS libraries were of low quality and excluded from the analyses. Furthermore, all nine samples were PCR positive for *Wolbachia* and *D. daci*^47^ (Table 1).

### Genome assembly

Sequence quality control and de novo assembly were performed in CLC Genomics Workbench as previously described^47^. Sequence identification and extraction was achieved by querying the reference genomes against the WGS library contig lists. First, BLASTn using the *M. australiensis* partial mitogenome (GenBank GU188852) was performed to filter the *D. daci* mitogenome from the contig list of *Bactrocera frauenfeldi* Bfra485 (Table 1). Contigs with the best hit were concatenated and manually gap-filled by iterative mapping of the trimmed reads at 90% similarity and 60–80% read length. The final *D. daci* draft mitogenome consensus sequence of this library was verified by mapping reads at 99% similarity. The final *D. daci* mitogenome extracted from the Bfra485 contig list was then used as a reference for the identification and filtration of *D. daci* mitogenomes from the other five libraries (Table 1). Similarly, BLASTn using the *Ceratitis capitata* mitogenome (GenBank AJ242872) was performed to identify and extract the fruit fly mitogenomes from the libraries, and the contigs with the best hit in each library were then assembled by iterative mapping as described earlier. The extracted *D. daci* and fruit fly mitogenomes were manually aligned and inspected in Geneious v10.0.9^73^.

### PCR amplification and Sanger sequencing of nad5

The *D. daci* mitogenome assembly revealed an unusual deletion of one nucleotide in *nad5*. This genomic dataset was obtained from WGS libraries which underwent REPLI-g amplification prior to library preparation^11^. To verify that this mutation was not due to a rare amplification error, PCR primers were designed to specifically amplify *nad5* of *D. daci* to confirm the WGS results, using Primer-BLAST (NCBI); Dd_nad5F: 5’ GAAACTGGAGTTGGAGCAGC 3’ and Dd_nad5R: 5’ ATAGCGTGTGATAAGTTAAATCGTT 3’ with an expected amplicon size of 396 bp. MyTaq™ Mix (Bioline) PCR reagents were used according to the manufacturer’s instructions. PCR cycling conditions began with an initial denaturation for 3 min at 94 °C, followed by 35 cycles of 30 s at 94 °C, 30 s at 50 °C and 1 min at 72 °C, then a final elongation step of 7 min at 72 °C. Five additional *D. daci* samples (Table S4) were PCR amplified and visualised by capillary electrophoresis on a QIAxcel system using a QIAxcel DNA screening kit (Qiagen). Prior to sequencing, PCR amplicons were treated with ExoSAP [exonuclease I (New England Biolabs, Ipswich, MA, USA) and shrimp alkaline phosphatase (Promega)] and incubated at 37 °C for 30 min, then 95 °C for 5 min. Sanger sequencing was performed using BigDye Terminator v3.1 kit (Applied Biosystems) and run on an Applied Biosystems 3500 Genetic Analyser.

### Mitogenome annotation and analysis

Annotation of the *D. daci* and fruit fly mitogenomes was performed using MITOS2 with “RefSeq 63 Metazoa” provided by MITOS2 and the invertebrate genetic code^74^, followed by manual verification of the coding regions and comparison with published mitochondrial sequences in Geneious v10.0.9 and NCBI BLASTn. The tRNA genes predicted by MITOS2 were confirmed using tRNAscan-SE^75^ and ARWEN^76^. The circular mitogenomes were visualised in Geneious v10.0.9. Comparative analyses of the composition skewness of the mitogenomes were calculated using the formulae: AT skew = [A − T]/[A + T] and GC skew = [G − C]/[G + C]. Comparative analysis of the mitogenomes codon usage was computed in MEGA7^77^.

### Comparative mitogenomics

Comparative analyses were performed using the six *D. daci* and nine fruit fly mitogenomes from this study, the mitogenomes of four other strepsipterans [*M. australiensis* (GU188852.1), *M. moldryzki* (JQ398619.1), *X. vesparum* (DQ364229.1) and *X. cf. moutoni* (MW222190)] and a representative member of other orders closely related to Strepsiptera including Coleoptera [*Tribolium castaneum* (AJ3124132)], Neuroptera [*D. pantherinus* (MK3012461)], Megaloptera [*Neochauliodes fraternus* (NC_0252821)], Raphidioptera [*Mongoloraphidia harmandi* (NC_0132511)] and Diptera [*B. tryoni* (NC_014611)].

### Intraspecific mitogenome diversity analyses

To determine the intraspecific genetic diversity across the *D. daci* mitogenome variants, we performed multiple sequence alignments of the six *D. daci* (Bfra485, Bn171, Bn342, Bt194, Bt210 and Zst503) mitogenome variants and used *D. daci* sequence information from the Bn240 library with a low mitogenome coverage which was insufficient for assembly but sufficient for SNP calling. Additionally, to compare the intraspecific genetic diversity in *D. daci* and the fruit fly host species, we performed individual multiple sequence alignments of 13 PCGs of the six assembled *D. daci*, five *B. neohumeralis* and three *B. tryoni* mitogenome variants (including *B. tryoni* NC_014611 obtained from GenBank). The multiple sequence alignments and DNA diversity analyses were performed using Geneious v10.0.9^73^ with default settings.

## Supplementary Information

Supplementary Information.

## Data Availability

Sample information is provided in Table 1, Table 2 and Table S3. Sequences were deposited in GenBank: the *D. daci* mitogenomes were highly similar (all SNPs are represented in Tables 2 and S5), and, therefore, only the *D. daci* mitogenome filtered from the WGS library of *B. frauenfeldi* 485 was deposited under accession number MW233588. Fruit fly mitogenomes from this study were deposited under accession numbers MZ520731- MZ520739. Raw reads for Bfra485, Bn171, Bn342, Bt194, Bt210 and Zst503 were submitted to NCBI Sequence Read Archive under the BioProject accession number PRJNA682518.
