# Supplementary material for: Substantial rearrangements, single nucleotide frameshift deletion and low diversity in mitogenome of Wolbachia-infected strepsipteran endoparasitoid in comparison to its tephritid hosts
Source: Sci Rep. 2022 Jan 10;12:477. doi: 10.1038/s41598-021-04398-y (PMC8748643; doi:10.1038/s41598-021-04398-y)
Supplement: Supplementary file 1 — Supplementary Information. [file 41598_2021_4398_MOESM1_ESM.docx]

**Figure S1:** Comparative analyses of the mitogenomes of Dipterophagus daci, tephritid fruit fly species and other reference species: (a) AT skew and (b) GC skew

**Table S1:** Summary of the mitogenome comparisons between Dipterophagus daci, tephritid fruit fly species and reference species, showing genome size, A+T%, AT-skew and GC-skew. Values of the newly sequenced mitogenomes are listed in bold. The SNPs of the D. daci mitogenomes are listed in Tables 2 and S5.

| **Species** | **Accession number** | **Order** | **Length** | | | | **A+T %** | | | | **Mitogenome** | **Mitogenome** |
| --- | --- | --- | --- | --- | --- | --- | --- | --- | --- | --- | --- | --- |
|  |  |  | **Mitogenome** | **PCGs** | **tRNAs** | **rRNAs** | **Mitogenome** | **PCGs** | **tRNAs** | **rRNAs** | **AT-skew** | **GC-skew** |
| ***Dipterophagus daci*_Bfra485** | **MW233588** | **Strepsiptera** | **16,255** | **10,696** | **1,424** | **2,074** | **84.7** | **82.5** | **86.2** | **87.2** | **0.0649** | **-0.3333** |
| ***Dipterophagus daci*_Bn171** |  |  | **16,248** | **10,696** | **1,424** | **2,074** | **84.4** | **82.5** | **85.9** | **87.2** | **0.0649** | **-0.3333** |
| ***Dipterophagus daci*_Bn342** |  |  | **16,243** | **10,696** | **1,424** | **2,074** | **84.3** | **82.5** | **86.2** | **87.2** | **0.0588** | **-0.3289** |
| ***Dipterophagus daci*_Bt194** |  |  | **16,247** | **10,696** | **1,424** | **2,074** | **84.7** | **82.5** | **86.2** | **87.2** | **0.0649** | **-0.3333** |
| ***Dipterophagus daci*_Bt210** |  |  | **16,247** | **10,696** | **1,424** | **2,075** | **83.3** | **82.5** | **86.2** | **87.2** | **0.0652** | **-0.3333** |
| ***Dipterophagus daci*_Zst503** |  |  | **16,248** | **10,696** | **1,424** | **2,074** | **84.7** | **82.5** | **86.2** | **87.2** | **0.0664** | **-0.3333** |
| *Mengenilla australiensis* | GU188852 |  | 13,421 | 10,736 | 1,046 | 1,632 | 84.3 | 83.8 | 86.7 | 85.9 | -0.0154 | -0.2692 |
| *Mengenilla moldryzki* | JQ398619 |  | 15,363 | 11,052 | 1,260 | 1,963 | 81.9 | 80.5 | 85.6 | 84.2 | -0.0256 | -0.3516 |
| *Xenos moutoni* | MW222190 |  | 16,717 | 10,663 | 1,379 | 1,969 | 82.5 | 79.8 | 83.8 | 83.4 | 0.052 | -0.3028 |
| *Xenos vesparum* | DQ364229 |  | 14,519 | 10,737 | 1,372 | 1,177 | 79.3 | 77.8 | 83.5 | 79.9 | 0.0921 | -0.2913 |
| ***Bactrocera frauenfeldi* 485** | **MZ520731** | **Diptera** | **15,935** | **11,189** | **1,468** | **2,081** | **74.1** | **71.7** | **75.1** | **77.5** | **0.0688** | **-0.2231** |
| ***Bactrocera neohumeralis* 135** | **MZ520732** |  | **15,924** | **11,185** | **1,467** | **2,087** | **72.5** | **69.6** | **75** | **77.5** | **0.0703** | **-0.2291** |
| ***Bactrocera neohumeralis* 171** | **MZ520733** |  | **15,927** | **11,188** | **1,467** | **2,087** | **72.5** | **69.7** | **74.9** | **77.6** | **0.0731** | **-0.2291** |
| ***Bactrocera neohumeralis* 240** | **MZ520734** |  | **15,922** | **11,188** | **1,467** | **2,087** | **72.4** | **69.7** | **74.9** | **77.6** | **0.0691** | **-0.2246** |
| ***Bactrocera neohumeralis* 244** | **MZ520735** |  | **15,927** | **11,188** | **1,467** | **2,087** | **72.5** | **69.7** | **74.9** | **77.6** | **0.0703** | **-0.2291** |
| ***Bactrocera neohumeralis* 342** | **MZ520736** |  | **15,923** | **11,188** | **1,467** | **2,087** | **72.4** | **69.7** | **74.9** | **77.6** | **0.0691** | **-0.2246** |
| ***Bactrocera tryoni* 194** | **MZ520737** |  | **15,926** | **11,189** | **1,467** | **2,110** | **72.3** | **69.4** | **75.4** | **77.3** | **0.0691** | **-0.2246** |
| ***Bactrocera tryoni* 210** | **MZ520738** |  | **15,925** | **11,186** | **1,467** | **2,110** | **72.4** | **69.6** | **75.3** | **77.5** | **0.0705** | **-0.2274** |
| *Bactrocera tryoni* | NC014611 |  | 15,925 | 11,187 | 1,467 | 2,115 | 72.5 | 69.6 | 75.3 | 77.5 | 0.0703 | -0.2246 |
| ***Zeugodacus strigifinis* 503** | **MZ520739** |  | **15,858** | **11,189** | **1,469** | **2,081** | **73.4** | **71.1** | **74.9** | **77.5** | **0.0845** | **-0.2434** |
| *Tribolium castaneum* | AJ312413 | Coleoptera | 15881 | 11091 | 1369 | 2041 | 71.7 | 69.2 | 75.4 | 75.7 | 0.1102 | -0.3074 |
| *Neochauliodes fraternus* | NC_025282 | Megaloptera | 15768 | 11092 | 1372 | 2092 | 77.3 | 75.4 | 76.8 | 80.7 | -0.0142 | -0.207 |
| *Dendroleon pantherinus* | MK301246 | Neuroptera | 15516 | 11158 | 1400 | 2087 | 73.2 | 72.2 | 75.4 | 77.7 | 0.071 | -0.1908 |
| *Mongoloraphidia harmandi* | NC_013251 | Raphidioptera | 16006 | 11100 | 1437 | 1602 | 80.3 | 78 | 81 | 80.7 | 0.0237 | -0.2347 |

**Table S2:** Annotation of the Dipterophagus daci mitogenome of Bfra485 (all other D. daci mitogenome variants are very similar, with SNPs listed in Table S5) and the tephritid fruit fly mitogenomes. Table shows, genes, gene location, gene length, intergenic sequences and the protein coding genes’ (PCGs) start and stop codons; + indicates genes coded on the major (leading) strand while - indicates genes encoded on the minor (lagging) strand; tRNA gene anticodons are shown in parentheses and positive values indicate intergenic nucleotides, negative values indicate overlaps and * indicates that TAA stop codon is presumably completed by addition of 3′A nucleotides to mRNA.

| ***Dipterophagus daci*_Bfra485** | | | | | |
| --- | --- | --- | --- | --- | --- |
| **Gene** | **Location** | **Strand** | **Length** | **Intergenic sequences** | **Start/stop**  **codon** |
| *trnI*(gat) | 1-65 | + | 65 | -3 |  |
| *trnQ*(ttg) | 63-132 | - | 70 | 6 |  |
| *trnM*(cat) | 139-202 | + | 64 | 0 |  |
| *nad2* | 203-1129 | + | 927 | -2 | ATA/TAA |
| *trnW*(tca) | 1128-1195 | + | 68 | 12 |  |
| *trnC*(gca) | 1208-1274 | - | 67 | 0 |  |
| *trnY*(gta) | 1274-1337 | - | 64 | 4 |  |
| *cox1* | 1342-2848 | + | 1507 | 0 | CAA/T* |
| *trnL_2_*(taa) | 2849-2910 | + | 62 | 9 |  |
| *cox2* | 2920-3571 | + | 652 | 0 | ATT/T* |
| *trnK*(ttt) | 3572-3634 | + | 63 | 2 |  |
| *trnD*(gtc) | 3637-3708 | + | 72 | 1 |  |
| *atp8* | 3710-3859 | + | 150 | -10 | ATT/TAA |
| *atp6* | 3850-4491 | + | 642 | 0 | ATG/TAA |
| *cox3* | 4491-5258 | + | 768 | 5 | ATG/TAA |
| *trnG*(tcc) | 5264-5323 | + | 60 | -3 |  |
| *nad3* | 5321-5663 | + | 343 | 0 | ATA/T* |
| *trnS_1_*(tct) | 5664-5724 | + | 61 | 15 |  |
| *trnR*(tcg) | 5740-5801 | + | 62 | 44 |  |
| *trnF*(gaa) | 5846-5907 | - | 62 | 25 |  |
| *trnN*(gtt) | 5933-5997 | + | 65 | -3 |  |
| *trnE*(ttc) | 5995-6059 | + | 65 | 5 |  |
| *trnA*(tgc) | 6065-6132 | + | 68 | -3 |  |
| *nad5_3'* | 6130-7479 | - | 1350 | -7 | ATT/TAA |
| *nad5_5'* | 7473-7763 | - | 291 | -3 | /TAA |
| *trnH*(gtg) | 7761-7822 | - | 62 | -2 |  |
| *nad4* | 7821-9083 | - | 1263 | 0 | ATG/T* |
| *nad4L* | 9083-9346 | - | 264 | 11 | ATA/TAA |
| *trnT*(tgt) | 9358-9421 | + | 64 | 6 |  |
| *trnP*(tgg) | 9428-9491 | - | 64 | 1 |  |
| *nad6* | 9493-9978 | + | 486 | 4 | ATT/TAA |
| *cob* | 9983-11093 | + | 1111 | 10 | ATG/T* |
| *trnL_1_*(tag) | 11104-11169 | - | 66 | 101 |  |
| *rrnS* | 11271-12064 | - | 794 | 34 |  |
| *nad1* | 12099-13040 | - | 942 | -3 | ATA/TAA |
| *rrnL* | 13038-14317 | - | 1280 | 0 |  |
| *trnV*(tac) | 14318-14380 | - | 63 | 0 |  |
| Control region | 14399-16180 | + | 1781 | gap |  |
| *trnS_2_*(tga) | 16181-16247 | + | 67 | 0 |  |

**Table S2 continued:**

| ***Bactrocera frauenfeldi* 485** | | | | | |
| --- | --- | --- | --- | --- | --- |
| **Gene** | **Location** | **Strand** | **Length** | **Intergenic sequence** | **Start/stop**  **codon** |
| *trnI*(gat) | 1-66 | + | 66 | -3 |  |
| *trnQ*(ttg) | 64-132 | - | 69 | 76 |  |
| *trnM*(cat) | 209-277 | + | 69 | 0 |  |
| *nad2* | 278-1300 | + | 1023 | 9 | ATT/TAA |
| *trnW*(tca) | 1310-1378 | + | 69 | -8 |  |
| *trnC*(gca) | 1371-1433 | - | 63 | 39 |  |
| *trnY*(gta) | 1473-1539 | - | 67 | -1 |  |
| *cox1* | 1538-3072 | + | 1535 | 0 | TCG/TA* |
| *trnL_2_*(taa) | 3073-3138 | + | 66 | 4 |  |
| *cox2* | 3143-3832 | + | 690 | 4 | ATG/TAA |
| *trnK*(ctt) | 3837-3907 | + | 71 | 2 |  |
| *trnD*(gtc) | 3910-3977 | + | 68 | 0 |  |
| *atp8* | 3978-4139 | + | 162 | -7 | GTG/TAA |
| *atp6* | 4133-4810 | + | 678 | 0 | ATG/TAA |
| *cox3* | 4810-5598 | + | 789 | 9 | ATG/TAA |
| *trnG*(tcc) | 5608-5672 | + | 65 | 0 |  |
| *nad3* | 5673-6026 | + | 354 | -2 | ATT/TAG |
| *trnA*(tgc) | 6025-6089 | + | 65 | 5 |  |
| *trnR*(tcg) | 6095-6158 | + | 64 | 26 |  |
| *trnN*(gtt) | 6185-6249 | + | 65 | 0 |  |
| *trnS_1_*(gct) | 6250-6317 | + | 68 | 0 |  |
| *trnE*(ttc) | 6318-6384 | + | 67 | 18 |  |
| *trnF*(gaa) | 6403-6467 | - | 65 | 0 |  |
| *nad5* | 6468-8187 | - | 1720 | 15 | ATT/T* |
| *trnH*(gtg) | 8203-8268 | - | 66 | 0 |  |
| *nad4* | 8269-9609 | - | 1341 | -7 | ATG/TAG |
| *nad4L* | 9603-9893 | - | 291 | 8 | ATG/TAA |
| *trnT*(tgt) | 9902-9966 | + | 65 | 0 |  |
| *trnP*(tgg) | 9967-10032 | - | 66 | 3 |  |
| *nad6* | 10035-10559 | + | 525 | 0 | ATT/TAA |
| *cob* | 10559-11693 | + | 1135 | 0 | ATG/T* |
| *trnS_2_*(tga) | 11694-11760 | + | 66 | 16 |  |
| *nad1* | 11776-12715 | - | 940 | 10 | ATA/T* |
| *trnL_1_*(tag) | 12726-12790 | - | 65 | 0 |  |
| *rrnL* | 12791-14119 | - | 1329 | 0 |  |
| *trnV*(tac) | 14120-14191 | - | 72 | -1 |  |
| *rrnS* | 14192-14983 | - | 792 | 0 |  |
| Control region | 14984-15579 | + | 595 | 0 |  |

**Table S2 continued:**

| ***Bactrocera neohumeralis* 135** | | | | | |
| --- | --- | --- | --- | --- | --- |
| **Gene** | **Location** | **Strand** | **Length** | **Intergenic sequence** | **Start/stop**  **codon** |
| *trnI*(gat) | 1-66 | + | 66 | -3 |  |
| *trnQ*(ttg) | 64-132 | - | 69 | 71 |  |
| *trnM*(cat) | 204-272 | + | 69 | 0 |  |
| *nad2* | 273-1295 | + | 1023 | 12 | ATT/TAA |
| *trnW*(tca) | 1308-1376 | + | 69 | -8 |  |
| *trnC*(gca) | 1369-1431 | - | 63 | 30 |  |
| *trnY*(gta) | 1462-1528 | - | 67 | -1 |  |
| *cox1* | 1527-3061 | + | 1535 | 0 | TCG/TA* |
| *trnL_2_*(taa) | 3062-3127 | + | 66 | 4 |  |
| *cox2* | 3132-3821 | + | 690 | 4 | ATG/TAA |
| *trnK*(ctt) | 3826-3896 | + | 71 | 0 |  |
| *trnD*(gtc) | 3897-3963 | + | 67 | 0 |  |
| *atp8* | 3964-4125 | + | 162 | -7 | GTG/TAA |
| *atp6* | 4119-4796 | + | 678 | 0 | ATG/TAA |
| *cox3* | 4796-5584 | + | 789 | 9 | ATG/TAA |
| *trnG*(tcc) | 5594-5658 | + | 65 | 0 |  |
| *nad3* | 5659-6009 | + | 351 | 2 | ATT/TAG |
| *trnA*(tgc) | 6011-6075 | + | 65 | 7 |  |
| *trnR*(tcg) | 6083-6146 | + | 64 | 34 |  |
| *trnN*(gtt) | 6180-6244 | + | 65 | 0 |  |
| *trnS_1_*(gct) | 6245-6312 | + | 68 | 0 |  |
| *trnE*(ttc) | 6313-6379 | + | 67 | 18 |  |
| *trnF*(gaa) | 6398-6462 | - | 65 | 0 |  |
| *nad5* | 6463-8182 | - | 1720 | 16 | ATT/T* |
| *trnH*(gtg) | 8198-8263 | - | 66 | 0 |  |
| *nad4* | 8264-9604 | - | 1341 | -7 | ATG/TAG |
| *nad4L* | 9598-9894 | - | 297 | 3 | ATG/TAA |
| *trnT*(tgt) | 9897-9961 | + | 65 | 0 |  |
| *trnP*(tgg) | 9962-10027 | - | 66 | 3 |  |
| *nad6* | 10030-10554 | + | 525 | 0 | ATT/TAA |
| *cob* | 10554-11688 | + | 1135 | 0 | ATG/T* |
| *trnS_2_*(tga) | 11689-11755 | + | 66 | 16 |  |
| *nad1* | 11771-12710 | - | 940 | 11 | ATT/T* |
| *trnL_1_*(tag) | 12721-12785 | - | 65 | 0 |  |
| *rrnL* | 12786-14082 | - | 1296 | 29 |  |
| *trnV*(tac) | 14112-14183 | - | 72 | 0 |  |
| *rrnS* | 14183-14972 | - | 790 | 0 |  |
| Control region | 14973-15567 | + | 594 | 0 |  |

**Table S2 continued:**

| ***Bactrocera neohumeralis* 244** | | | | | |
| --- | --- | --- | --- | --- | --- |
| **Gene** | **Location** | **Strand** | **Length** | **Intergenic sequence** | **Start/stop**  **codon** |
| *trnI*(gat) | 1-66 | + | 66 | -3 |  |
| *trnQ*(ttg) | 64-132 | - | 69 | 72 |  |
| *trnM*(cat) | 205-273 | + | 69 | 0 |  |
| *nad2* | 274-1296 | + | 1023 | 12 | ATT/TAA |
| *trnW*(tca) | 1309-1377 | + | 69 | -8 |  |
| *trnC*(gca) | 1370-1432 | - | 63 | 30 |  |
| *trnY*(gta) | 1463-1529 | - | 67 | -1 |  |
| *cox1* | 1528-3062 | + | 1535 | 0 | TCG/TA* |
| *trnL_2_*(taa) | 3063-3128 | + | 66 | 4 |  |
| *cox2* | 3133-3822 | + | 690 | 4 | ATG/TAA |
| *trnK*(ctt) | 3827-3897 | + | 71 | 2 |  |
| *trnD*(gtc) | 3900-3966 | + | 67 | 0 |  |
| *atp8* | 3967-4128 | + | 162 | -7 | GTG/TAA |
| *atp6* | 4122-4799 | + | 678 | 0 | ATG/TAA |
| *cox3* | 4799-5587 | + | 789 | 9 | ATG/TAA |
| *trnG*(tcc) | 5597-5661 | + | 65 | 0 |  |
| *nad3* | 5662-6015 | + | 354 | -2 | ATT/TAG |
| *trnA*(tgc) | 6014-6078 | + | 65 | 7 |  |
| *trnR*(tcg) | 6086-6149 | + | 64 | 33 |  |
| *trnN*(gtt) | 6183-6247 | + | 65 | 0 |  |
| *trnS_1_*(gct) | 6248-6315 | + | 68 | 0 |  |
| *trnE*(ttc) | 6316-6382 | + | 67 | 18 |  |
| *trnF*(gaa) | 6401-6465 | - | 65 | 0 |  |
| *nad5* | 6466-8185 | - | 1719 | 16 | ATT/T* |
| *trnH*(gtg) | 8201-8266 | - | 66 | 0 |  |
| *nad4* | 8267-9607 | - | 1341 | -7 | ATG/TAG |
| *nad4L* | 9601-9897 | - | 297 | 3 | ATG/TAA |
| *trnT*(tgt) | 9900-9964 | + | 65 | 0 |  |
| *trnP*(tgg) | 9965-10030 | - | 66 | 3 |  |
| *nad6* | 10033-10557 | + | 480 | 0 | ATT/TAA |
| *cob* | 10557-11691 | + | 1137 | 0 | ATG/T* |
| *trnS_2_*(tga) | 11692-11758 | + | 67 | 16 |  |
| *nad1* | 11774-12713 | - | 940 | 11 | ATT/T* |
| *trnL_1_*(tag) | 12724-12788 | - | 65 | 0 |  |
| *rrnL* | 12789-14085 | - | 1296 | 29 |  |
| *trnV*(tac) | 14115-14186 | - | 72 | 0 |  |
| *rrnS* | 14186-14975 | - | 790 | 0 |  |
| Control region | 14976-15570 | + | 594 | 0 |  |

**Table S2 continued:**

| ***Bactrocera neohumeralis* 171** | | | | | |
| --- | --- | --- | --- | --- | --- |
| **Gene** | **Location** | **Strand** | **Length** | **Intergenic sequence** | **Start/stop**  **codon** |
| *trnI*(gat) | 1-66 | + | 66 | -3 |  |
| *trnQ*(ttg) | 64-132 | - | 69 | 72 |  |
| *trnM*(cat) | 205-273 | + | 69 | 0 |  |
| *nad2* | 274-1296 | + | 1023 | 12 | ATT/TAA |
| *trnW*(tca) | 1309-1377 | + | 69 | -8 |  |
| *trnC*(gca) | 1370-1432 | - | 63 | 30 |  |
| *trnY*(gta) | 1463-1529 | - | 67 | -1 |  |
| *cox1* | 1528-3062 | + | 1535 | 0 | TCG/TA* |
| *trnL_2_*(taa) | 3063-3128 | + | 66 | 4 |  |
| *cox2* | 3133-3822 | + | 690 | 4 | ATG/TAA |
| *trnK*(ctt) | 3827-3897 | + | 71 | 2 |  |
| *trnD*(gtc) | 3900-3966 | + | 67 | 0 |  |
| *atp8* | 3967-4128 | + | 162 | -7 | GTG/TAA |
| *atp6* | 4122-4799 | + | 678 | 0 | ATG/TAA |
| *cox3* | 4799-5587 | + | 789 | 9 | ATG/TAA |
| *trnG*(tcc) | 5597-5661 | + | 65 | 0 |  |
| *nad3* | 5662-6015 | + | 354 | -2 | ATT/TAG |
| *trnA*(tgc) | 6014-6078 | + | 65 | 7 |  |
| *trnR*(tcg) | 6086-6149 | + | 64 | 33 |  |
| *trnN*(gtt) | 6183-6247 | + | 65 | 0 |  |
| *trnS_1_*(gct) | 6248-6315 | + | 68 | 0 |  |
| *trnE*(ttc) | 6316-6382 | + | 67 | 18 |  |
| *trnF*(gaa) | 6401-6465 | - | 65 | 0 |  |
| *nad5* | 6466-8185 | - | 1719 | 16 | ATT/T* |
| *trnH*(gtg) | 8201-8266 | - | 66 | 0 |  |
| *nad4* | 8267-9607 | - | 1341 | -7 | ATG/TAG |
| *nad4L* | 9601-9897 | - | 297 | 3 | ATG/TAA |
| *trnT*(tgt) | 9900-9964 | + | 65 | 0 |  |
| *trnP*(tgg) | 9965-10030 | - | 66 | 3 |  |
| *nad6* | 10033-10557 | + | 525 | 0 | ATT/TAA |
| *cob* | 10557-11691 | + | 1135 | 0 | ATG/T* |
| *trnS_2_*(tga) | 11692-11758 | + | 67 | 16 |  |
| *nad1* | 11774-12713 | - | 940 | 11 | ATT/T* |
| *trnL_1_*(tag) | 12724-12788 | - | 65 | 0 |  |
| *rrnL* | 12789-14085 | - | 1320 | 29 |  |
| *trnV*(tac) | 14115-14186 | - | 72 | 0 |  |
| *rrnS* | 14186-14975 | - | 790 | 0 |  |
| Control region | 14976-15570 | + | 597 | 0 |  |

**Table S2 continued:**

| ***Bactrocera neohumeralis* 240** | | | | | |
| --- | --- | --- | --- | --- | --- |
| **Gene** | **Location** | **Strand** | **Length** | **Intergenic sequence** | **Start/stop**  **codon** |
| *trnI*(gat) | 1-66 | + | 66 | -3 |  |
| *trnQ*(ttg) | 64-132 | - | 69 | 71 |  |
| *trnM*(cat) | 204-272 | + | 69 | 0 |  |
| *nad2* | 273-1295 | + | 1023 | 12 | ATT/TAA |
| *trnW*(tca) | 1308-1376 | + | 69 | -8 |  |
| *trnC*(gca) | 1369-1431 | - | 63 | 30 |  |
| *trnY*(gta) | 1462-1528 | - | 67 | -1 |  |
| *cox1* | 1527-3061 | + | 1535 | 0 | TCG/TA* |
| *trnL_2_*(taa) | 3062-3127 | + | 66 | 4 |  |
| *cox2* | 3132-3821 | + | 690 | 4 | ATG/TAA |
| *trnK*(ctt) | 3826-3896 | + | 71 | 0 |  |
| *trnD*(gtc) | 3897-3963 | + | 67 | 0 |  |
| *atp8* | 3964-4125 | + | 162 | -4 | GTG/TAA |
| *atp6* | 4122-4796 | + | 675 | 0 | ATA/TAA |
| *cox3* | 4796-5584 | + | 789 | 9 | ATG/TAA |
| *trnG*(tcc) | 5594-5658 | + | 65 | 0 |  |
| *nad3* | 5659-6012 | + | 354 | -2 | ATT/TAG |
| *trnA*(tgc) | 6011-6075 | + | 65 | 7 |  |
| *trnR*(tcg) | 6083-6146 | + | 64 | 34 |  |
| *trnN*(gtt) | 6180-6244 | + | 65 | 0 |  |
| *trnS_1_*(gct) | 6245-6312 | + | 68 | 0 |  |
| *trnE*(ttc) | 6313-6379 | + | 67 | 18 |  |
| *trnF*(gaa) | 6398-6462 | - | 65 | 0 |  |
| *nad5* | 6463-8182 | - | 1719 | 16 | ATT/T* |
| *trnH*(gtg) | 8198-8263 | - | 66 | 0 |  |
| *nad4* | 8264-9604 | - | 1341 | -7 | ATG/TAG |
| *nad4L* | 9598-9894 | - | 297 | 3 | ATG/TAA |
| *trnT*(tgt) | 9897-9961 | + | 65 | 0 |  |
| *trnP*(tgg) | 9962-10027 | - | 66 | 3 |  |
| *nad6* | 10030-10554 | + | 525 | 0 | ATT/TAA |
| *cob* | 10554-11688 | + | 1135 | 0 | ATG/T* |
| *trnS_2_*(tga) | 11689-11755 | + | 67 | 16 |  |
| *nad1* | 11771-12710 | - | 940 | 11 | ATT/T* |
| *trnL_1_*(tag) | 12721-12785 | - | 65 | 0 |  |
| *rrnL* | 12786-14082 | - | 1320 | 29 |  |
| *trnV*(tac) | 14112-14183 | - | 72 | 0 |  |
| *rrnS* | 14183-14972 | - | 790 | 0 |  |
| Control region | 14973-15566 | + | 594 | 0 |  |

**Table S2 continued:**

| ***Bactrocera neohumeralis* 342** | | | | | |
| --- | --- | --- | --- | --- | --- |
| **Gene** | **Location** | **Strand** | **Length** | **Intergenic sequence** | **Start/stop**  **codon** |
| *trnI*(gat) | 1-66 | + | 66 | -3 |  |
| *trnQ*(ttg) | 64-132 | - | 69 | 71 |  |
| *trnM*(cat) | 204-272 | + | 69 | 0 |  |
| *nad2* | 273-1295 | + | 1023 | 12 | ATT/TAA |
| *trnW*(tca) | 1308-1376 | + | 69 | -8 |  |
| *trnC*(gca) | 1369-1431 | - | 63 | 30 |  |
| *trnY*(gta) | 1462-1528 | - | 67 | -1 |  |
| *cox1* | 1527-3061 | + | 1535 | 0 | TCG/TA* |
| *trnL_2_*(taa) | 3062-3127 | + | 66 | 4 |  |
| *cox2* | 3132-3821 | + | 690 | 4 | ATG/TAA |
| *trnK*(ctt) | 3826-3896 | + | 71 | 0 |  |
| *trnD*(gtc) | 3897-3963 | + | 67 | 0 |  |
| *atp8* | 3964-4125 | + | 162 | -4 | GTG/TAA |
| *atp6* | 4119-4796 | + | 678 | 0 | ATA/TAA |
| *cox3* | 4796-5584 | + | 789 | 9 | ATG/TAA |
| *trnG*(tcc) | 5594-5658 | + | 65 | 0 |  |
| *nad3* | 5659-6012 | + | 354 | -2 | ATT/TAG |
| *trnA*(tgc) | 6011-6075 | + | 65 | 7 |  |
| *trnR*(tcg) | 6083-6146 | + | 64 | 34 |  |
| *trnN*(gtt) | 6180-6244 | + | 65 | 0 |  |
| *trnS_1_*(gct) | 6245-6312 | + | 68 | 0 |  |
| *trnE*(ttc) | 6313-6379 | + | 67 | 18 |  |
| *trnF*(gaa) | 6398-6462 | - | 65 | 0 |  |
| *nad5* | 6463-8182 | - | 1719 | 16 | ATT/T* |
| *trnH*(gtg) | 8198-8263 | - | 66 | 0 |  |
| *nad4* | 8264-9604 | - | 1341 | -7 | ATG/TAG |
| *nad4L* | 9598-9894 | - | 297 | 3 | ATG/TAA |
| *trnT*(tgt) | 9897-9961 | + | 65 | 0 |  |
| *trnP*(tgg) | 9962-10027 | - | 66 | 3 |  |
| *nad6* | 10030-10554 | + | 525 | 0 | ATT/TAA |
| *cob* | 10554-11688 | + | 1135 | 0 | ATG/T* |
| *trnS_2_*(tga) | 11689-11755 | + | 66 | 16 |  |
| *nad1* | 11771-12710 | - | 940 | 11 | ATT/T* |
| *trnL_1_*(tag) | 12721-12785 | - | 65 | 0 |  |
| *rrnL* | 12763-14082 | - | 1320 | 29 |  |
| *trnV*(tac) | 14112-14183 | - | 72 | 0 |  |
| *rrnS* | 14183-14972 | - | 790 | 0 |  |
| Control region | 14973-15566 | + | 594 | 0 |  |

**Table S2 continued:**

| ***Bactrocera tryoni* 194** | | | | | |
| --- | --- | --- | --- | --- | --- |
| **Gene** | **Location** | **Strand** | **Length** | **Intergenic sequence** | **Start/stop**  **codon** |
| *trnI*(gat) | 1-66 | + | 66 | -3 |  |
| *trnQ*(ttg) | 64-132 | - | 69 | 71 |  |
| *trnM*(cat) | 204-272 | + | 69 | 0 |  |
| *nad2* | 273-1295 | + | 1023 | 12 | ATT/TAA |
| *trnW*(tca) | 1308-1376 | + | 69 | -8 |  |
| *trnC*(gca) | 1369-1431 | - | 63 | 30 |  |
| *trnY*(gta) | 1462-1528 | - | 67 | -1 |  |
| *cox1* | 1527-3061 | + | 1535 | 0 | TCG/TA* |
| *trnL_2_*(taa) | 3062-3127 | + | 66 | 4 |  |
| *cox2* | 3132-3821 | + | 690 | 4 | ATG/TAA |
| *trnK*(ctt) | 3826-3896 | + | 71 | 0 |  |
| *trnD*(gtc) | 3899-3965 | + | 67 | 0 |  |
| *atp8* | 3966-4127 | + | 162 | -4 | GTG/TAA |
| *atp6* | 4121-4798 | + | 678 | 0 | ATA/TAA |
| *cox3* | 4798-5586 | + | 789 | 9 | ATG/TAA |
| *trnG*(tcc) | 5596-5660 | + | 65 | 0 |  |
| *nad3* | 5661-6014 | + | 354 | -2 | ATT/TAG |
| *trnA*(tgc) | 6013-6077 | + | 65 | 7 |  |
| *trnR*(tcg) | 6085-6148 | + | 64 | 34 |  |
| *trnN*(gtt) | 6182-6246 | + | 65 | 0 |  |
| *trnS_1_*(gct) | 6247-6314 | + | 68 | 0 |  |
| *trnE*(ttc) | 6315-6381 | + | 67 | 18 |  |
| *trnF*(gaa) | 6400-6464 | - | 65 | 0 |  |
| *nad5* | 6465-8184 | - | 1719 | 16 | ATT/T* |
| *trnH*(gtg) | 8200-8265 | - | 66 | 0 |  |
| *nad4* | 8266-9606 | - | 1341 | -7 | ATG/TAG |
| *nad4L* | 9600-9896 | - | 297 | 3 | ATG/TAA |
| *trnT*(tgt) | 9899-9963 | + | 65 | 0 |  |
| *trnP*(tgg) | 9964-10029 | - | 66 | 3 |  |
| *nad6* | 10032-10556 | + | 525 | 0 | ATT/TAA |
| *cob* | 10556-11690 | + | 1135 | 0 | ATG/T* |
| *trnS_2_*(tga) | 11691-11757 | + | 67 | 16 |  |
| *nad1* | 11773-12712 | - | 940 | 11 | ATT/T* |
| *trnL_1_*(tag) | 12723-12787 | - | 65 | 0 |  |
| *rrnL* | 12765-14084 | - | 1320 | 29 |  |
| *trnV*(tac) | 14114-14185 | - | 72 | 0 |  |
| *rrnS* | 14185-14974 | - | 790 | 0 |  |
| Control region | 14975-15569 | + | 594 | 0 |  |

**Table S2 continued:**

| ***Bactrocera tryoni* 210** | | | | | |
| --- | --- | --- | --- | --- | --- |
| **Gene** | **Location** | **Strand** | **Length** | **Intergenic sequence** | **Start/stop**  **codon** |
| *trnI*(gat) | 1-66 | + | 66 | -3 |  |
| *trnQ*(ttg) | 64-132 | - | 69 | 71 |  |
| *trnM*(cat) | 204-272 | + | 69 | 0 |  |
| *nad2* | 273-1295 | + | 1023 | 12 | ATT/TAA |
| *trnW*(tca) | 1308-1376 | + | 69 | -8 |  |
| *trnC*(gca) | 1369-1431 | - | 63 | 30 |  |
| *trnY*(gta) | 1462-1528 | - | 67 | -1 |  |
| *cox1* | 1527-3061 | + | 1535 | 0 | TCG/TA* |
| *trnL_2_*(taa) | 3062-3127 | + | 66 | 4 |  |
| *cox2* | 3132-3821 | + | 690 | 4 | ATG/TAA |
| *trnK*(ctt) | 3826-3896 | + | 71 | 0 |  |
| *trnD*(gtc) | 3898-3964 | + | 67 | 0 |  |
| *atp8* | 3965-4126 | + | 162 | -4 | GTG/TAA |
| *atp6* | 4120-4797 | + | 678 | 0 | ATA/TAA |
| *cox3* | 4797-5585 | + | 789 | 9 | ATG/TAA |
| *trnG*(tcc) | 5595-5659 | + | 65 | 0 |  |
| *nad3* | 5660-6013 | + | 354 | -2 | ATT/TAG |
| *trnA*(tgc) | 6012-6076 | + | 65 | 7 |  |
| *trnR*(tcg) | 6084-6147 | + | 64 | 34 |  |
| *trnN*(gtt) | 6181-6245 | + | 65 | 0 |  |
| *trnS_1_*(gct) | 6246-6313 | + | 68 | 0 |  |
| *trnE*(ttc) | 6314-6379 | + | 66 | 18 |  |
| *trnF*(gaa) | 6398-6462 | - | 65 | 0 |  |
| *nad5* | 6463-8182 | - | 1719 | 16 | ATT/T* |
| *trnH*(gtg) | 8198-8263 | - | 66 | 0 |  |
| *nad4* | 8264-9604 | - | 1341 | -7 | ATG/TAG |
| *nad4L* | 9598-9894 | - | 297 | 3 | ATG/TAA |
| *trnT*(tgt) | 9897-9961 | + | 65 | 0 |  |
| *trnP*(tgg) | 9962-10027 | - | 66 | 3 |  |
| *nad6* | 10030-10552 | + | 523 | 0 | ATT/TAA |
| *cob* | 10554-11688 | + | 1135 | 0 | ATG/T* |
| *trnS_2_*(tga) | 11689-11755 | + | 67 | 16 |  |
| *nad1* | 11691-12608 | - | 940 | 11 | ATT/T* |
| *trnL_1_*(tag) | 12721-12785 | - | 65 | 0 |  |
| *rrnL* | 12763-14082 | - | 1320 | 29 |  |
| *trnV*(tac) | 14112-14183 | - | 72 | 0 |  |
| *rrnS* | 14183-14972 | - | 790 | 0 |  |
| Control region | 14973-15568 | + | 594 | 0 |  |

**Table S2 continued:**

| ***Zeugodacus strigifinis* 503** | | | | | |
| --- | --- | --- | --- | --- | --- |
| **Gene** | **Location** | **Strand** | **Length** | **Intergenic sequence** | **Start/stop**  **codon** |
| *trnI*(gat) | 1-65 | + | 65 | -3 |  |
| *trnQ*(ttg) | 63-131 | - | 69 | 7 |  |
| *trnM*(cat) | 139-207 | + | 69 | 0 |  |
| *nad2* | 208-1230 | + | 1023 | 11 | ATT/TAA |
| *trnW*(tca) | 1241-1308 | + | 68 | 36 |  |
| *trnC*(gca) | 1345-1407 | - | 63 | 0 |  |
| *trnY*(gta) | 1408-1474 | - | 67 | -1 |  |
| *cox1* | 1473-3007 | + | 1535 | 0 | TCG/TA* |
| *trnL_2_*(taa) | 3007-3072 | + | 66 | 5 |  |
| *cox2* | 3078-3767 | + | 690 | 4 | ATG/TAA |
| *trnK*(ctt) | 3772-3842 | + | 71 | -1 |  |
| *trnD*(gtc) | 3842-3910 | + | 69 | 0 |  |
| *atp8* | 3911-4072 | + | 162 | -7 | ATT/TAA |
| *atp6* | 4066-4743 | + | 678 | 0 | ATG/TAA |
| *cox3* | 4743-5531 | + | 789 | 6 | ATG/TAA |
| *trnG*(tcc) | 5538-5602 | + | 65 | 0 |  |
| *nad3* | 5603-5956 | + | 354 | -2 | ATT/TAG |
| *trnA*(tgc) | 5955-6020 | + | 66 | 0 |  |
| *trnR*(tcg) | 6021-6084 | + | 64 | 34 |  |
| *trnN*(gtt) | 6119-6183 | + | 65 | 0 |  |
| *trnS_1_*(gct) | 6184-6251 | + | 68 | 0 |  |
| *trnE*(ttc) | 6252-6319 | + | 68 | 18 |  |
| *trnF*(gaa) | 6338-6402 | - | 65 | 0 |  |
| *nad5* | 6403-8122 | - | 1720 | 16 | ATT/T* |
| *trnH*(gtg) | 8138-8203 | - | 66 | 0 |  |
| *nad4* | 8203-9543 | - | 1341 | -7 | ATG/TAA |
| *nad4L* | 9537-9833 | - | 297 | 2 | ATG/TAA |
| *trnT*(tgt) | 9836-9900 | + | 65 | 0 |  |
| *trnP*(tgg) | 9901-9966 | - | 66 | 3 |  |
| *nad6* | 9969-10493 | + | 525 | 0 | ATT/TAA |
| *cob* | 10493-11627 | + | 1135 | 0 | ATG/T* |
| *trnS_2_*(tga) | 11628-11694 | + | 67 | 16 |  |
| *nad1* | 11710-12649 | - | 940 | 11 | ATT/T* |
| *trnL_1_*(tag) | 12659-12724 |  | 66 | 0 |  |
| *rrnL* | 12725-14016 | - | 1292 | 37 |  |
| *trnV*(tac) | 14053-14124 | - | 72 | 0 |  |
| *rrnS* | 14124-14912 | - | 789 | 0 |  |
| Control region | 14913-15502 | + | 589 | 0 |  |

**Table S3:** Comparative analysis of the mitogenome relative synonymous codon usage (RSCU) of the mitogenomes of *Dipterophagus daci*, tephritid fruit fly species and reference species. Newly sequenced mitogenomes are listed in bold.

| **Species** | **Accession number** | **UUU(F)** | **UUC(F)** | **UUA(L)** | **UUG(L)** | **CUU(L)** | **CUC(L)** | **CUA(L)** | **CUG(L)** | **AUU(I)** | **AUC(I)** | **AUA(M)** | **AUG(M)** | **GUU(V)** | **GUC(V)** |
| --- | --- | --- | --- | --- | --- | --- | --- | --- | --- | --- | --- | --- | --- | --- | --- |
| ***Dipterophagus daci*_Bfra485** | **MW233588** | 1.9 | 0.1 | 4.96 | 0.17 | 0.34 | 0.06 | 0.44 | 0.02 | 1.8 | 0.2 | 1.8 | 0.2 | 1.93 | 0.15 |
| ***Dipterophagus daci*_Bn171** |  | 1.89 | 0.11 | 4.95 | 0.17 | 0.34 | 0.07 | 0.45 | 0.02 | 1.79 | 0.21 | 1.8 | 0.2 | 2.08 | 0.15 |
| ***Dipterophagus daci*_Bn342** |  | 1.9 | 0.1 | 4.95 | 0.17 | 0.35 | 0.06 | 0.45 | 0.02 | 1.79 | 0.21 | 1.81 | 0.19 | 2.02 | 0.16 |
| ***Dipterophagus daci*_Bt194** |  | 1.9 | 0.1 | 4.96 | 0.17 | 0.33 | 0.07 | 0.45 | 0.02 | 1.79 | 0.21 | 1.81 | 0.19 | 2.02 | 0.16 |
| ***Dipterophagus daci*_Bt210** |  | 1.9 | 0.1 | 4.95 | 0.17 | 0.34 | 0.07 | 0.45 | 0.02 | 1.79 | 0.21 | 1.81 | 0.19 | 2.02 | 0.16 |
| ***Dipterophagus daci*_Zst503** |  | 1.9 | 0.1 | 4.96 | 0.17 | 0.33 | 0.07 | 0.45 | 0.02 | 1.79 | 0.21 | 1.81 | 0.19 | 2.02 | 0.16 |
| *Mengenilla australiensis* | GU188852 | 1.92 | 0.08 | 5.22 | 0.16 | 0.42 | 0.03 | 0.17 | 0 | 1.93 | 0.07 | 1.86 | 0.14 | 2.2 | 0.05 |
| *Mengenilla moldryzki* | JQ398619 | 1.75 | 0.25 | 4.45 | 0.28 | 0.62 | 0.12 | 0.53 | 0.01 | 1.77 | 0.23 | 1.85 | 0.15 | 1.44 | 0.19 |
| *Xenos vesparum* | DQ364229 | 1.71 | 0.29 | 3.9 | 0.38 | 0.58 | 0.12 | 0.95 | 0.07 | 1.71 | 0.29 | 1.77 | 0.23 | 1.85 | 0.08 |
| *Xenos moutoni* | MW222190 | 1.55 | 0.45 | 3.19 | 0.56 | 0.92 | 0.39 | 0.78 | 0.16 | 1.55 | 0.45 | 1.7 | 0.3 | 1.21 | 0.42 |
| ***Bactrocera frauenfeldi*485** | **MZ520731** | 1.55 | 0.45 | 3.76 | 0.52 | 0.64 | 0.08 | 0.89 | 0.1 | 1.8 | 0.2 | 1.68 | 0.32 | 1.71 | 0.19 |
| ***Bactrocera neohumeralis*135** | **MZ520732** | 1.51 | 0.49 | 3.24 | 0.73 | 0.77 | 0.06 | 1.07 | 0.13 | 1.69 | 0.31 | 1.56 | 0.44 | 1.7 | 0.23 |
| ***Bactrocera neohumeralis*171** | **MZ520733** | 1.53 | 0.47 | 3.26 | 0.72 | 0.76 | 0.07 | 1.04 | 0.15 | 1.68 | 0.32 | 1.58 | 0.42 | 1.68 | 0.23 |
| ***Bactrocera neohumeralis*240** | **MZ520734** | 1.5 | 0.5 | 3.24 | 0.71 | 0.77 | 0.06 | 1.09 | 0.13 | 1.69 | 0.31 | 1.57 | 0.43 | 1.69 | 0.25 |
| ***Bactrocera neohumeralis*244** | **MZ520735** | 1.5 | 0.5 | 3.31 | 0.7 | 0.75 | 0.08 | 0.98 | 0.17 | 1.69 | 0.31 | 1.56 | 0.44 | 1.7 | 0.22 |
| ***Bactrocera neohumeralis*342** | **MZ520736** | 1.51 | 0.49 | 3.21 | 0.75 | 0.76 | 0.07 | 1.09 | 0.12 | 1.7 | 0.3 | 1.57 | 0.43 | 1.74 | 0.21 |
| ***Bactrocera tryoni*194** | **MZ520737** | 1.51 | 0.49 | 3.26 | 0.73 | 0.76 | 0.07 | 1.06 | 0.12 | 1.71 | 0.29 | 1.55 | 0.45 | 1.69 | 0.25 |
| ***Bactrocera tryoni*210** | **MZ520738** | 1.49 | 0.51 | 3.26 | 0.73 | 0.77 | 0.06 | 1.05 | 0.13 | 1.68 | 0.32 | 1.59 | 0.41 | 1.71 | 0.23 |
| *Bactrocera tryoni* | NC0146111 | 1.52 | 0.48 | 3.21 | 0.75 | 0.78 | 0.06 | 1.06 | 0.14 | 1.7 | 0.3 | 1.59 | 0.41 | 1.69 | 0.25 |
| ***Zeugodacus strigifinis* 503** | **MZ520739** | 1.32 | 0.68 | 2.31 | 0.73 | 0.95 | 0.45 | 1.24 | 0.32 | 1.45 | 0.55 | 1.57 | 0.43 | 1.44 | 0.56 |
| *Tribolium castaneum* | AJ312413 | 1.4 | 0.6 | 2.57 | 0.84 | 0.99 | 0.37 | 1.11 | 0.13 | 1.6 | 0.4 | 1.58 | 0.42 | 1.73 | 0.22 |
| *Neochauliodes fraternus* | NC_025282 | 1.84 | 0.16 | 4.79 | 0.14 | 0.68 | 0.08 | 0.28 | 0.02 | 1.88 | 0.12 | 1.82 | 0.18 | 2.06 | 0.18 |
| *Dendroleon pantherinus* | MK301246 | 1.59 | 0.41 | 3.94 | 0.57 | 0.61 | 0.12 | 0.67 | 0.09 | 1.72 | 0.28 | 1.77 | 0.23 | 1.87 | 0.1 |
| *Mongoloraphidia harmandi* | NC_013251 | 1.78 | 0.22 | 4.87 | 0.26 | 0.41 | 0.03 | 0.4 | 0.03 | 1.82 | 0.18 | 1.94 | 0.06 | 2.01 | 0.19 |

**Table S3 continued:**

| **Species** | **Accession number** | **GUA(V)** | **GUG(V)** | **UCU(S)** | **UCC(S)** | **UCA(S)** | **UCG(S)** | **CCU(P)** | **CCC(P)** | **CCA(P)** | **CCG(P)** | **ACU(T)** | **ACC(T)** | **ACA(T)** | **ACG(T)** |
| --- | --- | --- | --- | --- | --- | --- | --- | --- | --- | --- | --- | --- | --- | --- | --- |
| ***Dipterophagus daci*_Bfra485** | **MW233588** | 1.45 | 0.47 | 2.27 | 0.15 | 1.94 | 0.05 | 2 | 0.4 | 1.47 | 0.13 | 1.94 | 0.26 | 1.76 | 0.03 |
| ***Dipterophagus daci*_Bn171** |  | 1.38 | 0.38 | 2.28 | 0.16 | 1.96 | 0.05 | 2 | 0.4 | 1.47 | 0.13 | 1.96 | 0.27 | 1.75 | 0.03 |
| ***Dipterophagus daci*_Bn342** |  | 1.44 | 0.39 | 2.28 | 0.16 | 1.96 | 0.05 | 2 | 0.4 | 1.47 | 0.13 | 1.96 | 0.27 | 1.75 | 0.03 |
| ***Dipterophagus daci*_Bt194** |  | 1.44 | 0.39 | 2.28 | 0.16 | 1.96 | 0.05 | 2 | 0.4 | 1.47 | 0.13 | 1.96 | 0.27 | 1.75 | 0.03 |
| ***Dipterophagus daci*_Bt210** |  | 1.44 | 0.39 | 2.27 | 0.16 | 1.95 | 0.05 | 2 | 0.4 | 1.47 | 0.13 | 1.96 | 0.27 | 1.75 | 0.03 |
| ***Dipterophagus daci*_Zst503** |  | 1.44 | 0.39 | 2.28 | 0.16 | 1.96 | 0.05 | 2 | 0.4 | 1.47 | 0.13 | 1.96 | 0.27 | 1.75 | 0.03 |
| *Mengenilla australiensis* | GU188852 | 1.76 | 0 | 2.27 | 0.25 | 2.22 | 0.03 | 2.69 | 0.12 | 1.18 | 0 | 2.14 | 0.12 | 1.7 | 0.04 |
| *Mengenilla moldryzki* | JQ398619 | 2.07 | 0.3 | 2.2 | 0.37 | 2.23 | 0.12 | 2.06 | 0.39 | 1.55 | 0 | 2.21 | 0.35 | 1.4 | 0.04 |
| *Xenos vesparum* | DQ364229 | 1.62 | 0.45 | 1.63 | 0.37 | 2.01 | 0.07 | 1.81 | 0.87 | 1.25 | 0.08 | 1.32 | 0.57 | 2.08 | 0.03 |
| *Xenos moutoni* | MW222190 | 1.74 | 0.63 | 1.72 | 1.24 | 1.81 | 0.29 | 1.66 | 1.06 | 1.15 | 0.13 | 1.53 | 0.96 | 1.27 | 0.24 |
| ***Bactrocera frauenfeldi*485** | **MZ520731** | 1.84 | 0.26 | 2.56 | 0.44 | 2.15 | 0.17 | 2.39 | 0.47 | 1.05 | 0.09 | 1.64 | 0.4 | 1.92 | 0.04 |
| ***Bactrocera neohumeralis*135** | **MZ520732** | 1.72 | 0.34 | 2.6 | 0.36 | 2.17 | 0.17 | 2.01 | 0.79 | 1.05 | 0.15 | 1.55 | 0.53 | 1.71 | 0.22 |
| ***Bactrocera neohumeralis*171** | **MZ520733** | 1.77 | 0.32 | 2.63 | 0.34 | 2.19 | 0.17 | 2.03 | 0.79 | 1.12 | 0.06 | 1.57 | 0.53 | 1.67 | 0.24 |
| ***Bactrocera neohumeralis*240** | **MZ520734** | 1.72 | 0.34 | 2.6 | 0.36 | 2.17 | 0.17 | 2.01 | 0.79 | 1.05 | 0.15 | 1.56 | 0.53 | 1.69 | 0.22 |
| ***Bactrocera neohumeralis*244** | **MZ520735** | 1.72 | 0.36 | 2.62 | 0.34 | 2.16 | 0.19 | 2.09 | 0.74 | 1.06 | 0.12 | 1.57 | 0.53 | 1.67 | 0.24 |
| ***Bactrocera neohumeralis*342** | **MZ520736** | 1.67 | 0.37 | 2.62 | 0.34 | 2.16 | 0.17 | 1.99 | 0.82 | 1.08 | 0.12 | 1.56 | 0.53 | 1.69 | 0.22 |
| ***Bactrocera tryoni*194** | **MZ520737** | 1.7 | 0.36 | 2.61 | 0.36 | 2.18 | 0.17 | 1.99 | 0.82 | 1.08 | 0.12 | 1.56 | 0.53 | 1.69 | 0.22 |
| ***Bactrocera tryoni*210** | **MZ520738** | 1.66 | 0.39 | 2.55 | 0.41 | 2.17 | 0.17 | 1.96 | 0.88 | 1.05 | 0.12 | 1.56 | 0.53 | 1.71 | 0.2 |
| *Bactrocera tryoni* | NC0146111 | 1.72 | 0.34 | 2.64 | 0.34 | 2.16 | 0.17 | 1.97 | 0.82 | 1.09 | 0.12 | 1.52 | 0.57 | 1.73 | 0.18 |
| ***Zeugodacus strigifinis* 503** | **MZ520739** | 1.72 | 0.28 | 1.55 | 0.88 | 1.82 | 0.46 | 1.18 | 1.11 | 1.34 | 0.36 | 1.21 | 1.05 | 1.39 | 0.35 |
| *Tribolium castaneum* | AJ312413 | 1.65 | 0.4 | 2.1 | 0.51 | 2.23 | 0.38 | 1.58 | 0.69 | 1.49 | 0.24 | 1.45 | 0.7 | 1.69 | 0.15 |
| *Neochauliodes fraternus* | NC_025282 | 1.63 | 0.14 | 2.61 | 0.36 | 2.07 | 0.07 | 2.58 | 0.34 | 1.02 | 0.06 | 1.86 | 0.48 | 1.66 | 0 |
| *Dendroleon pantherinus* | MK301246 | 1.81 | 0.22 | 1.96 | 0.4 | 2.43 | 0.15 | 1.91 | 0.65 | 1.14 | 0.31 | 1.64 | 0.4 | 1.85 | 0.11 |
| *Mongoloraphidia harmandi* | NC_013251 | 1.71 | 0.08 | 2.99 | 0.23 | 1.6 | 0.09 | 2.7 | 0.41 | 0.89 | 0 | 2.14 | 0.41 | 1.41 | 0.05 |

**Table S3 continued:**

| **Species** | **Accession number** | **GCU(A)** | **GCC(A)** | **GCA(A)** | **GCG(A)** | **UAU(Y)** | **UAC(Y)** | **UAA(*)** | **UAG(*)** | **CAU(H)** | **CAC(H)** | **CAA(Q)** | **CAG(Q)** | **AAU(N)** | **AAC(N)** |
| --- | --- | --- | --- | --- | --- | --- | --- | --- | --- | --- | --- | --- | --- | --- | --- |
| ***Dipterophagus daci*_Bfra485** | **MW233588** | 2.31 | 0.38 | 1.23 | 0.08 | 1.71 | 0.29 | 2 | 0 | 1.57 | 0.43 | 2 | 0 | 1.6 | 0.4 |
| ***Dipterophagus daci*_Bn171** |  | 2.35 | 0.39 | 1.25 | 0 | 1.73 | 0.27 | 2 | 0 | 1.52 | 0.48 | 2 | 0 | 1.59 | 0.41 |
| ***Dipterophagus daci*_Bn342** |  | 2.35 | 0.39 | 1.25 | 0 | 1.72 | 0.28 | 2 | 0 | 1.57 | 0.43 | 2 | 0 | 1.59 | 0.41 |
| ***Dipterophagus daci*_Bt194** |  | 2.35 | 0.39 | 1.25 | 0 | 1.72 | 0.28 | 2 | 0 | 1.57 | 0.43 | 2 | 0 | 1.59 | 0.41 |
| ***Dipterophagus daci*_Bt210** |  | 2.35 | 0.39 | 1.25 | 0 | 1.72 | 0.28 | 2 | 0 | 1.57 | 0.43 | 2 | 0 | 1.59 | 0.41 |
| ***Dipterophagus daci*_Zst503** |  | 2.35 | 0.39 | 1.25 | 0 | 1.72 | 0.28 | 2 | 0 | 1.57 | 0.43 | 2 | 0 | 1.59 | 0.41 |
| *Mengenilla australiensis* | GU188852 | 2.4 | 0.4 | 1.2 | 0 | 1.93 | 0.07 | 1.82 | 0.18 | 1.9 | 0.1 | 2 | 0 | 1.94 | 0.06 |
| *Mengenilla moldryzki* | JQ398619 | 2.31 | 0.38 | 1.31 | 0 | 1.74 | 0.26 | 1.38 | 0.62 | 1.62 | 0.38 | 1.81 | 0.19 | 1.79 | 0.21 |
| *Xenos vesparum* | DQ364229 | 2.16 | 0.74 | 1.11 | 0 | 1.74 | 0.26 | 1.85 | 0.15 | 1.61 | 0.39 | 1.65 | 0.35 | 1.71 | 0.29 |
| *Xenos moutoni* | MW222190 | 1.71 | 1.03 | 1.14 | 0.11 | 1.65 | 0.35 | 1.78 | 0.22 | 1.42 | 0.58 | 1.51 | 0.49 | 1.66 | 0.34 |
| ***Bactrocera frauenfeldi*485** | **MZ520731** | 2.12 | 0.41 | 1.4 | 0.06 | 1.58 | 0.42 | 1.23 | 0.77 | 1.25 | 0.75 | 1.73 | 0.27 | 1.68 | 0.32 |
| ***Bactrocera neohumeralis*135** | **MZ520732** | 1.95 | 0.72 | 1.19 | 0.14 | 1.48 | 0.52 | 1.23 | 0.77 | 0.99 | 1.01 | 1.63 | 0.37 | 1.51 | 0.49 |
| ***Bactrocera neohumeralis*171** | **MZ520733** | 1.92 | 0.74 | 1.18 | 0.16 | 1.52 | 0.48 | 1.23 | 0.77 | 1.05 | 0.95 | 1.68 | 0.32 | 1.48 | 0.52 |
| ***Bactrocera neohumeralis*240** | **MZ520734** | 1.91 | 0.74 | 1.21 | 0.14 | 1.47 | 0.53 | 1.23 | 0.77 | 1.01 | 0.99 | 1.65 | 0.35 | 1.51 | 0.49 |
| ***Bactrocera neohumeralis*244** | **MZ520735** | 1.9 | 0.76 | 1.18 | 0.16 | 1.51 | 0.49 | 1.23 | 0.77 | 1.04 | 0.96 | 1.65 | 0.35 | 1.49 | 0.51 |
| ***Bactrocera neohumeralis*342** | **MZ520736** | 1.88 | 0.72 | 1.28 | 0.12 | 1.46 | 0.54 | 1.23 | 0.77 | 0.99 | 1.01 | 1.65 | 0.35 | 1.51 | 0.49 |
| ***Bactrocera tryoni*194** | **MZ520737** | 1.9 | 0.74 | 1.22 | 0.14 | 1.49 | 0.51 | 1.23 | 0.77 | 0.99 | 1.01 | 1.65 | 0.35 | 1.51 | 0.49 |
| ***Bactrocera tryoni*210** | **MZ520738** | 1.89 | 0.74 | 1.19 | 0.18 | 1.47 | 0.53 | 1.23 | 0.77 | 0.99 | 1.01 | 1.65 | 0.35 | 1.49 | 0.51 |
| *Bactrocera tryoni* | NC0146111 | 1.92 | 0.71 | 1.24 | 0.12 | 1.49 | 0.51 | 1.23 | 0.77 | 0.99 | 1.01 | 1.63 | 0.37 | 1.51 | 0.49 |
| ***Zeugodacus strigifinis* 503** | **MZ520739** | 1.13 | 1.46 | 1.3 | 0.11 | 1.44 | 0.56 | 1.49 | 0.51 | 1.4 | 0.6 | 1.3 | 0.7 | 1.47 | 0.53 |
| *Tribolium castaneum* | AJ312413 | 1.42 | 0.93 | 1.5 | 0.15 | 1.38 | 0.62 | 1.54 | 0.46 | 1.05 | 0.95 | 1.51 | 0.49 | 1.31 | 0.69 |
| *Neochauliodes fraternus* | NC_025282 | 2.3 | 0.46 | 1.15 | 0.09 | 1.66 | 0.34 | 1.8 | 0.2 | 1.76 | 0.24 | 1.78 | 0.22 | 1.84 | 0.16 |
| *Dendroleon pantherinus* | MK301246 | 2.14 | 0.34 | 1.41 | 0.11 | 1.39 | 0.61 | 1.67 | 0.33 | 1.42 | 0.58 | 1.74 | 0.26 | 1.63 | 0.37 |
| *Mongoloraphidia harmandi* | NC_013251 | 2.25 | 0.41 | 1.27 | 0.06 | 1.69 | 0.31 | 1.85 | 0.15 | 1.63 | 0.37 | 1.87 | 0.13 | 1.73 | 0.27 |

**Table S3 continued:**

| **Species** | **Accession number** | **AAA(K)** | **AAG(K)** | **GAU(D)** | **GAC(D)** | **GAA(E)** | **GAG(E)** | **UGU(C)** | **UGC(C)** | **UGA(W)** | **UGG(W)** | **CGU(R)** | **CGC(R)** | **CGA(R)** | **CGG(R)** |
| --- | --- | --- | --- | --- | --- | --- | --- | --- | --- | --- | --- | --- | --- | --- | --- |
| ***Dipterophagus daci*_Bfra485** | **MW233588** | 1.86 | 0.14 | 1.64 | 0.36 | 1.83 | 0.17 | 1.63 | 0.37 | 1.9 | 0.1 | 1.33 | 0.12 | 2.42 | 0.12 |
| ***Dipterophagus daci*_Bn171** |  | 1.88 | 0.12 | 1.61 | 0.39 | 1.83 | 0.17 | 1.62 | 0.38 | 1.92 | 0.08 | 1.25 | 0.12 | 2.5 | 0.12 |
| ***Dipterophagus daci*_Bn342** |  | 1.88 | 0.12 | 1.61 | 0.39 | 1.83 | 0.17 | 1.62 | 0.38 | 1.92 | 0.08 | 1.25 | 0.12 | 2.5 | 0.12 |
| ***Dipterophagus daci*_Bt194** |  | 1.88 | 0.12 | 1.61 | 0.39 | 1.83 | 0.17 | 1.62 | 0.38 | 1.92 | 0.08 | 1.25 | 0.12 | 2.5 | 0.12 |
| ***Dipterophagus daci*_Bt210** |  | 1.88 | 0.12 | 1.61 | 0.39 | 1.83 | 0.17 | 1.62 | 0.38 | 1.92 | 0.08 | 1.25 | 0.12 | 2.5 | 0.12 |
| ***Dipterophagus daci*_Zst503** |  | 1.88 | 0.12 | 1.61 | 0.39 | 1.83 | 0.17 | 1.62 | 0.38 | 1.92 | 0.08 | 1.25 | 0.12 | 2.5 | 0.12 |
| *Mengenilla australiensis* | GU188852 | 1.97 | 0.03 | 1.63 | 0.37 | 1.82 | 0.18 | 1.92 | 0.08 | 1.94 | 0.06 | 1.33 | 0.21 | 2.36 | 0.1 |
| *Mengenilla moldryzki* | JQ398619 | 1.77 | 0.23 | 1.83 | 0.17 | 1.57 | 0.43 | 1.83 | 0.17 | 1.86 | 0.14 | 1.54 | 0.1 | 1.74 | 0.62 |
| *Xenos vesparum* | DQ364229 | 1.72 | 0.28 | 1.57 | 0.43 | 1.4 | 0.6 | 1.76 | 0.24 | 1.89 | 0.11 | 1.38 | 0.5 | 1.63 | 0.5 |
| *Xenos moutoni* | MW222190 | 1.77 | 0.23 | 1.62 | 0.38 | 1.62 | 0.38 | 1.4 | 0.6 | 1.54 | 0.46 | 1.33 | 0.19 | 1.52 | 0.95 |
| ***Bactrocera frauenfeldi*485** | **MZ520731** | 1.3 | 0.7 | 1.51 | 0.49 | 1.87 | 0.13 | 1.95 | 0.05 | 1.7 | 0.3 | 1.17 | 0.28 | 2.14 | 0.41 |
| ***Bactrocera neohumeralis*135** | **MZ520732** | 1.31 | 0.69 | 1.26 | 0.74 | 1.81 | 0.19 | 1.72 | 0.28 | 1.66 | 0.34 | 0.98 | 0.21 | 2.32 | 0.49 |
| ***Bactrocera neohumeralis*171** | **MZ520733** | 1.26 | 0.74 | 1.34 | 0.66 | 1.73 | 0.27 | 1.81 | 0.19 | 1.72 | 0.28 | 1.05 | 0.14 | 2.25 | 0.56 |
| ***Bactrocera neohumeralis*240** | **MZ520734** | 1.31 | 0.69 | 1.29 | 0.71 | 1.79 | 0.21 | 1.72 | 0.28 | 1.62 | 0.38 | 0.98 | 0.21 | 2.32 | 0.49 |
| ***Bactrocera neohumeralis*244** | **MZ520735** | 1.29 | 0.71 | 1.23 | 0.77 | 1.79 | 0.21 | 1.67 | 0.33 | 1.7 | 0.3 | 1.05 | 0.14 | 2.32 | 0.49 |
| ***Bactrocera neohumeralis*342** | **MZ520736** | 1.33 | 0.67 | 1.3 | 0.7 | 1.76 | 0.24 | 1.71 | 0.29 | 1.66 | 0.34 | 0.98 | 0.21 | 2.39 | 0.42 |
| ***Bactrocera tryoni*194** | **MZ520737** | 1.31 | 0.69 | 1.26 | 0.74 | 1.79 | 0.21 | 1.77 | 0.23 | 1.66 | 0.34 | 0.98 | 0.21 | 2.32 | 0.49 |
| ***Bactrocera tryoni*210** | **MZ520738** | 1.31 | 0.69 | 1.26 | 0.74 | 1.79 | 0.21 | 1.81 | 0.19 | 1.66 | 0.34 | 0.98 | 0.21 | 2.39 | 0.42 |
| *Bactrocera tryoni* | NC0146111 | 1.36 | 0.64 | 1.32 | 0.68 | 1.73 | 0.27 | 1.77 | 0.23 | 1.68 | 0.32 | 0.98 | 0.21 | 2.46 | 0.35 |
| ***Zeugodacus strigifinis* 503** | **MZ520739** | 1.56 | 0.44 | 1.29 | 0.71 | 1.24 | 0.76 | 1.29 | 0.71 | 1.43 | 0.57 | 0.55 | 1.03 | 1.45 | 0.97 |
| *Tribolium castaneum* | AJ312413 | 1.33 | 0.67 | 1.38 | 0.63 | 1.6 | 0.4 | 1.48 | 0.52 | 1.84 | 0.16 | 1.14 | 0.21 | 2.29 | 0.36 |
| *Neochauliodes fraternus* | NC_025282 | 1.57 | 0.43 | 1.69 | 0.31 | 1.83 | 0.17 | 1.88 | 0.12 | 1.84 | 0.16 | 1.53 | 0.22 | 2.04 | 0.22 |
| *Dendroleon pantherinus* | MK301246 | 1.58 | 0.42 | 1.65 | 0.35 | 1.59 | 0.41 | 1.83 | 0.17 | 1.64 | 0.36 | 1.61 | 0.07 | 2.11 | 0.21 |
| *Mongoloraphidia harmandi* | NC_013251 | 1.79 | 0.21 | 1.78 | 0.22 | 1.84 | 0.16 | 1.82 | 0.18 | 1.91 | 0.09 | 1.28 | 0.16 | 2.4 | 0.16 |

**Table S3 continued:**

| **Species** | **Accession number** | **AGU(S)** | **AGC(S)** | **AGA(S)** | **AGG(S)** | **GGU(G)** | **GGC(G)** | **GGA(G)** | **GGG(G)** |
| --- | --- | --- | --- | --- | --- | --- | --- | --- | --- |
| ***Dipterophagus daci*_Bfra485** | **MW233588** | 0.8 | 0.15 | 2.27 | 0.36 | 1.09 | 0.27 | 1.86 | 0.78 |
| ***Dipterophagus daci*_Bn171** |  | 0.79 | 0.16 | 2.25 | 0.34 | 1.1 | 0.27 | 1.88 | 0.75 |
| ***Dipterophagus daci*_Bn342** |  | 0.79 | 0.16 | 2.25 | 0.34 | 1.1 | 0.27 | 1.88 | 0.75 |
| ***Dipterophagus daci*_Bt194** |  | 0.79 | 0.16 | 2.25 | 0.34 | 1.1 | 0.27 | 1.88 | 0.75 |
| ***Dipterophagus daci*_Bt210** |  | 0.82 | 0.16 | 2.24 | 0.34 | 1.1 | 0.27 | 1.88 | 0.75 |
| ***Dipterophagus daci*_Zst503** |  | 0.79 | 0.16 | 2.25 | 0.34 | 1.1 | 0.27 | 1.88 | 0.75 |
| *Mengenilla australiensis* | GU188852 | 0.77 | 0.03 | 2.17 | 0.25 | 1.09 | 0.09 | 2.54 | 0.28 |
| *Mengenilla moldryzki* | JQ398619 | 0.63 | 0.19 | 2.04 | 0.23 | 1.04 | 0.26 | 1.88 | 0.81 |
| *Xenos vesparum* | DQ364229 | 0.73 | 0.26 | 2.36 | 0.57 | 1.13 | 0.44 | 1.83 | 0.6 |
| *Xenos moutoni* | MW222190 | 0.53 | 0.57 | 1.29 | 0.55 | 1.28 | 0.64 | 1.28 | 0.8 |
| ***Bactrocera frauenfeldi*485** | **MZ520731** | 1.29 | 0.15 | 1.24 | 0 | 1.01 | 0.12 | 2.09 | 0.78 |
| ***Bactrocera neohumeralis*135** | **MZ520732** | 1.13 | 0.31 | 1.25 | 0 | 0.82 | 0.14 | 2.04 | 1 |
| ***Bactrocera neohumeralis*171** | **MZ520733** | 1.04 | 0.39 | 1.25 | 0 | 0.88 | 0.12 | 1.96 | 1.04 |
| ***Bactrocera neohumeralis*240** | **MZ520734** | 1.11 | 0.34 | 1.25 | 0 | 0.82 | 0.14 | 1.99 | 1.05 |
| ***Bactrocera neohumeralis*244** | **MZ520735** | 1.03 | 0.38 | 1.27 | 0 | 0.87 | 0.14 | 2.07 | 0.92 |
| ***Bactrocera neohumeralis*342** | **MZ520736** | 1.08 | 0.36 | 1.27 | 0 | 0.84 | 0.14 | 1.92 | 1.1 |
| ***Bactrocera tryoni*194** | **MZ520737** | 1.14 | 0.29 | 1.26 | 0 | 0.82 | 0.14 | 1.97 | 1.07 |
| ***Bactrocera tryoni*210** | **MZ520738** | 1.06 | 0.41 | 1.23 | 0 | 0.82 | 0.16 | 1.9 | 1.12 |
| *Bactrocera tryoni* | NC0146111 | 1.06 | 0.38 | 1.25 | 0 | 0.84 | 0.12 | 1.92 | 1.12 |
| ***Zeugodacus strigifinis* 503** | **MZ520739** | 0.95 | 0.73 | 1 | 0.62 | 0.91 | 0.73 | 1.39 | 0.97 |
| *Tribolium castaneum* | AJ312413 | 0.55 | 0.09 | 1.88 | 0.27 | 0.68 | 0.31 | 2.23 | 0.77 |
| *Neochauliodes fraternus* | NC_025282 | 0.97 | 0.21 | 1.69 | 0.02 | 1.38 | 0.09 | 1.93 | 0.6 |
| *Dendroleon pantherinus* | MK301246 | 1.22 | 0.3 | 1.52 | 0.02 | 1.4 | 0.19 | 1.4 | 1.02 |
| *Mongoloraphidia harmandi* | NC_013251 | 0.88 | 0.02 | 2.18 | 0 | 0.91 | 0.08 | 2.58 | 0.43 |

**Table S4**: Summary of the *Dipterophagus daci* samples used for amplification and sequencing of the *nad5* gene. Table shows the sample, sample ID, collection locality and collection year.

| **Sample** | **Sample ID** | **Collection locality** | **Collection year** |
| --- | --- | --- | --- |
| *Dipterophagus daci* male | Dd45 | Cairns | 2019/2020 |
| *Dipterophagus daci* male pupa | Dd10 | Cairns | 2019/2020 |
| *Dipterophagus daci* male pupa | Dd57 | Cairns | 2019/2020 |
| *Dipterophagus daci* male pupa | Dd55 | Airlie Beach | 2019/2020 |
| *Dipterophagus daci* male pupa | Dd1 | Townsville | 2019/2020 |

**Table S5:** Intraspecific mitogenome diversity of *Dipterophagus daci*, listed by the collection locality, *Wolbachia* infection status (+ or -) with *w*Ddac1 (ST-285) and *w*Ddac2 (ST-289), and the single nucleotide polymorphism (SNP) position in the mitogenome. The * denotes the assembled reference genome of *D. daci* from Bfra485 (MW233588), ^ denotes library with low coverage that did not allow assembly of the mitogenome and — denotes a nucleotide deletion and the # denotes the non-coding region. Empty cells are indicative of no difference of a mitogenome variant from the reference genome of *D. daci* from Bfra485.

| **Collection locality** | ***w*Ddac1** | ***w*Ddac2** | **Gene** | ***cox1*** | | **#** | ***nad5*** | | | | ***nad4*** | | ***cob*** | | **#** | | | | ***rrnS*** | |
| --- | --- | --- | --- | --- | --- | --- | --- | --- | --- | --- | --- | --- | --- | --- | --- | --- | --- | --- | --- | --- |
|  |  |  | **Nucleotide position in the mitogenome** | **1,762** | **2,546** | **3,738** | **6,408** | **6,607** | **6,912** | **7,306** | **7,869** | **8,640** | **10,276** | **11,033** | **11,207** | **11,208** | **11,209** | **11,209** | **11,837** | **11,983** |
| **Cairns** | **y** | **y** | ***Dipterophagus daci*_Bfra485*** | **C** | **A** | **C** | **G** | **G** | **T** | **C** | **T** | **G** | **A** | **A** | **A** | **A** | **T** | **A** | **G** | **—** |
| Townsville | y | y | *Dipterophagus daci*_Bn171 | T |  |  |  |  | C |  | C | A | C | G |  |  |  |  | A | A |
| Mourilyan Harbour | y | n | *Dipterophagus daci*_Bn240^ |  |  | T |  |  |  | G | C | A | C |  | — | — | — | — | A | A |
| Mackay | y | y | *Dipterophagus daci*_Bn342 |  |  |  |  | A |  |  |  |  |  |  | — | — | — | — | A | A |
| Cairns | y | y | *Dipterophagus daci*_Bt194 |  |  |  | A |  |  |  |  |  |  |  |  |  |  |  |  |  |
| Mackay | y | y | *Dipterophagus daci*_Bt210 |  | G |  |  |  |  |  |  |  |  |  | — | — | — | — | A | A |
| Cairns | y | y | *Dipterophagus daci*_Zst503 |  |  |  | A |  |  |  |  |  |  |  |  |  |  |  |  |  |

**Table S5 continued:**

| **Collection locality** | ***w*Ddac1** | ***w*Ddac2** | **Gene** | ***rrnL*** | **control region** | | | | | | | | | | | | | | | |
| --- | --- | --- | --- | --- | --- | --- | --- | --- | --- | --- | --- | --- | --- | --- | --- | --- | --- | --- | --- | --- |
|  |  |  | **Nucleotide position in the mitogenome** | **13,064** | **14,967** | **15,000** | **15,074** | **15,075** | **15,078** | **15,089** | **15,135** | **15,564** | **15,667** | **15,668** | **15,752** | **15,764** | **15,043** | **15,044** | **15,045** | **16,070** |
| **Cairns** | **y** | **y** | ***Dipterophagus daci*_Bfra485*** | **A** | **G** | **C** | **G** | **T** | **A** | **T** | **G** | **G** | **—** | **—** | **T** | **C** | **T** | **A** | — | **A** |
| Townsville | y | y | *Dipterophagus daci*_Bn171 |  | T |  |  |  |  | C | A | A |  |  |  | T |  |  |  |  |
| Mourilyan Harbour | y | n | *Dipterophagus daci*_Bn240^ | G |  |  |  |  |  |  |  |  |  |  |  |  |  |  |  |  |
| Mackay | y | y | *Dipterophagus daci*_Bn342 |  |  | A | T | A | T |  |  |  |  |  | A |  | A | — |  |  |
| Cairns | y | y | *Dipterophagus daci*_Bt194 |  |  |  |  |  |  |  |  |  |  |  |  |  |  |  |  |  |
| Mackay | y | y | *Dipterophagus daci*_Bt210 |  |  |  |  |  |  |  |  |  | T | A |  |  | A |  | A |  |
| Cairns | y | y | *Dipterophagus daci*_Zst503 |  |  |  |  |  |  |  |  |  |  |  |  |  | A |  | A | A |
